# Osteoarthritis: molecular pathogenesis and potential therapeutic options

**DOI:** 10.1038/s41392-025-02556-6

**Published:** 2026-03-04

**Authors:** Yi Zhang, Yanqi Han, Ying Sun, Longhui Hao, Yue Gao, Jun Ye, Hongliang Wang, Tiantai Zhang, Yuling Liu, Yanfang Yang

**Affiliations:** 1 State Key Laboratory of Bioactive Substance and Function of Natural Medicines, Institute of Materia Medica, Chinese Academy of Medical Sciences & Peking Union Medical College, Beijing, China; 2 Beijing Key Laboratory of Key Technologies for Natural Drug Delivery and Novel Formulations, Institute of Materia Medica, Chinese Academy of Medical Sciences & Peking Union Medical College, Beijing, China

**Keywords:** Rheumatic diseases, Inflammation, Drug delivery, Nanobiotechnology, Rheumatic diseases

## Abstract

Osteoarthritis (OA) is a debilitating joint disorder that causes chronic pain, inflammation, and detrimental bone alterations. Despite significant advances in understanding OA pathogenesis, current therapeutic strategies remain inadequate in halting disease progression or providing effective pain relief, highlighting unmet clinical needs. Recent insights into OA nociceptive pathways, inflammatory mediators, and organelle dysfunction have revealed promising therapeutic targets. Specifically, OA progression is driven by mitochondrial dysfunction (marked by accumulated damaged mitochondria with excessive ROS production and impaired ATP synthesis), lysosomal destabilization (due to persistent hydroxyapatite digestion causing acidification loss, membrane permeabilization, and chondrocyte apoptosis), and unresolved ER stress (resulting from compensatory protein overproduction that exacerbates cartilage degradation). In this review, we aim to provide a comprehensive exploration of the nociceptive pathways linking the knee joint to the central nervous system, shedding light on the mechanisms underlying OA-associated pain. We further analyzed pathological changes in bone architecture and chondrocytes, emphasizing the synergistic roles of inflammatory cytokines and organelle-specific dysfunctions. Building on these mechanistic insights, we delineate emerging pharmacological strategies designed to concurrently address inflammatory cascades, restore organelle homeostasis (via mitophagy potentiation, lysosomal integrity preservation, and ER stress alleviation), and attenuate nociceptive signaling—thereby establishing a multimodal therapeutic paradigm to ameliorate both structural degeneration and clinical manifestations of OA. We also highlight advanced organelle-targeted drug delivery systems designed to increase the therapeutic efficacy and stability of these treatments. Collectively, these advancements provide a framework for novel OA interventions.

## Introduction

Osteoarthritis (OA) is a chronic, debilitating joint disorder characterized by widespread morphological alterations, inflammation, and intolerable hyperalgesia. In 2019, China reported the highest number of OA cases globally, with 132.81 million individuals affected, representing a dramatic 156.58% increase since 1990.^1^ This growing prevalence constitutes a significant and escalating public health challenge, underscoring the urgent need for more effective clinical interventions.

Current clinical therapies for OA are primarily palliative because of the complex nature of its pathogenesis. Standard treatments are generally categorized into physiotherapies, pharmacological approaches, and surgical procedures, on the basis of the modality of intervention. Nonpharmacological strategies include weight management, exercise, assistive devices, and various physical therapies (e.g., therapeutic ultrasound, electrical stimulation, phototherapy, hydrotherapy, magnetotherapy, cryotherapy, and thermotherapy), along with acupuncture.^2^ Although these treatments can alleviate pain and improve joint mobility to some extent, they are predominantly symptomatic and fail to halt disease progression.

Pharmacological therapies, as outlined by international guidelines, typically involve oral nonsteroidal anti-inflammatory drugs (NSAIDs), intra-articular hyaluronic acid (HA) injections, and opioid administration.^1,2^ While these treatments may offer anti-inflammatory or analgesic benefits for certain patients, they often fall short of providing sustained symptomatic relief in those with moderate to advanced OA.^3^ Total joint replacement is considered the final therapeutic option when conservative treatments fail to yield improvements.^4^ Although clinical data demonstrate significant enhancements in joint function, pain relief, and quality of life following surgery,^5^ challenges remain—such as the emergence of catastrophizing pain at multiple sites during postsurgical rehabilitation and the limited lifespan of joint prostheses, which may require revision surgery.^6^ Consequently, novel, effective pharmacological therapies with less invasive administration and fewer adverse events have emerged as a growing clinical demand for OA treatment.

OA has a complicated and multifaceted pathology involving inflammation, hyperalgesia, and cartilage breakdown. Pain, a primary reason that OA patients seek medical treatment, is poorly managed by existing analgesics, highlighting the need for a deeper understanding of OA pain mechanisms. Disease pathogenesis is primarily attributed to pathological changes in three aspects: the extracellular matrix (ECM), chondrocytes, and homeostasis between osteoblasts and osteoclasts. Among these factors, inflammation driven by cytokines and disturbances in chondrocyte organelle function have been identified as key contributing factors. Recent research suggests that targeting cytokine signaling and organelle dysfunction in chondrocytes may offer promising therapeutic opportunities.

This review systematically examines the mechanisms underlying OA pain, along with the roles of cytokines and chondrocyte organelles in disease progression. On the basis of these insights, we discuss emerging therapeutic targets and corresponding pharmacological agents or drug delivery systems that may not only slow but also potentially reverse OA progression while simultaneously delivering meaningful symptomatic relief.

## Epidemiology of osteoarthritis

Recent data from the Global Burden of Disease (GBD) study indicate that OA affected 7.6% of the global population—~595 million individuals—in 2020.^7,8^ The increasing prevalence of OA is partly attributed to geographic region, national economic status, and sociodemographic variables.^7,9,10^ Emerging evidence from global studies suggests that individuals in socioeconomically deprived areas experience higher rates of OA (hand, hip, and knee).^11,12^ Interestingly, countries with a high sociodemographic index (SDI), such as Australia, also report increased OA incidence, whereas middle-SDI countries such as China face a rapidly escalating disease burden.^7,13^

At the individual level, OA incidence demonstrates considerable heterogeneity across demographics. In 2020, the global age-standardized prevalence revealed marked sex disparities, with women exhibiting 8,058.9 cases per 100,000 (95% UI 7251.9–8867.9) compared with 5780.1 per 100,000 (95% UI 5217.8–6341.2) in men.^7^ Age further stratifies this disparity, with the prevalence increasing sharply among older populations and peaking between the ages of 55 and 64.^14^ Additionally, OA epidemiology is influenced by ethnic disparities.^10,15^ US-based research indicates that African Americans report more severe pain and disability than White Americans do (WOMAC SMD: 0.57, 95% CI: 0.54–0.61), whereas Asian Americans also report higher pain levels than their White counterparts do.^10,16^

OA susceptibility is further influenced by key biomechanical and lifestyle factors. Obesity is one of the most significant modifiable risk factors, particularly for knee OA, with elevated body mass index (BMI) accounting for ~20% of incident cases.^7,17^ Previous traumatic joint injuries substantially increase OA risk; for example, an isolated anterior cruciate ligament (ACL) injury increases the likelihood of OA by 4.2 times (95% CI: 3.8–10.5), whereas an isolated meniscal injury increases the risk by 6.3-fold (95% CI: 4.9–8.3).^18^ Physical activity shows a biphasic association with OA risk: both sedentary lifestyles and frequent, high-intensity exercise are linked to greater incidence, whereas moderate activity appears protective.^9^ Furthermore, individuals in physically demanding occupations—such as construction workers, floor layers, bricklayers, fishermen, and farmers—are disproportionately affected by chronic mechanical stress on joints.^19^

## Osteoarthritis prevention

OA prevention strategies are generally classified into primary and secondary prevention strategies. Primary prevention aims to reduce behaviors or exposures that lead to disease, thereby preventing its onset. In contrast, secondary prevention focuses on halting the progression of disease among individuals already exposed to risk factors.^20,21^

In OA, three key modifiable risk factors stand out: obesity, prior knee injury, and excessive musculoskeletal loading.^22,23^ Obesity is particularly important, with a normal BMI defined as 18.5–25 kg/m² and obesity defined as a BMI over 30 kg/m².^24^ Maintaining a BMI below 25 kg/m² is estimated to reduce the population-attributable risk of OA by 27–53%.^22^ Weight-reduction strategies center on healthy eating and consistent physical activity.^23^ While dietary restriction can aid in weight loss, macronutrients (protein, carbohydrate, and fat) have a limited impact unless an individual has obesity-related comorbidities such as diabetes or cardiovascular disease.^21^ Moreover, sustaining weight loss through diet alone proves challenging—studies have shown that up to 50% of individuals regain some weight loss within a year.^25,26^ Exercise alone is less effective for weight loss, but it is essential for weight maintenance.^21,27^ Therefore, a combined approach involving both diet and exercise is generally recommended. Other interventions include cognitive behavioral therapy, which is associated with substantial weight reduction, and bariatric surgery, which offers the most effective and sustained weight loss outcomes.^21^

Previous knee injuries are also strong predictors of OA.^28^ Epidemiological studies have revealed that one in two adults who suffer severe knee injury will develop OA in that joint during their lifetime. More than 50% of ACL-injured knees progress to OA within 10 years.^29^ However, consistent adherence to neuromuscular and proprioceptive training—typically 15–20 min per session, two to three times per week—has been shown to reduce the incidence of ACL injury by up to 50% in high-risk populations such as adolescents and adult athletes.^21,22,30^ Nevertheless, epidemiological data indicate that injury rates revert to baseline levels upon discontinuation of these programs.^21^

For individuals with preexisting risk factors such as knee injuries or physically demanding occupations, additional behavioral modifications—including the avoidance of occupational squatting, kneeling, and heavy lifting—can reduce OA risk by 15% to 30% in men.^22^ Notably, surgical repair of knee injuries does not mitigate the increased OA risk caused by initial trauma, as evidenced by longitudinal biomechanical studies.^30,31^ Current clinical guidelines emphasize secondary injury prevention and joint-protective exercise regimens as foundational therapeutic strategies.^30^

## Osteoarthritis diagnosis

Timely and accurate differentiation of symptomatic knee OA from other causes of knee pain is critical for ensuring appropriate patient management in clinical practice. When pain or discomfort lasts for weeks to months and is accompanied by the progressive worsening of symptoms—such as stiffness and limited mobility—with minimal pain-free intervals, this clinical pattern is typically indicative of early-stage knee OA.^32^ Clinical examination often reveals pain upon mobilization, joint-line tenderness, crepitus, or mild joint effusion, and these findings are supported by features such as older age, high BMI, a history of knee trauma, or a family history of OA.^33^ However, the elusive nature of typical radiographic features in early-stage OA has led to the absence of validated diagnostic criteria to date.^34^

The diagnosis of symptomatic OA is largely dependent on physical examination. In patients with knee OA, knee effusion is commonly absent or minimal and normothermic, accompanied by popliteal or “baker” cysts—extensions of synovial swelling that can be palpated at the posterior aspect of the knee—and may present with valgus or varus deformities. In contrast, patients with inflammatory, infectious, or crystalline arthritis typically present with a warm knee and easily palpable effusion.^35^ Additionally, the presence of Heberden’s and Bouchard’s nodes—swelling in the distal and proximal interphalangeal joints, respectively—is indicative of polyarticular disease and is linked to the progression of knee OA.^34,36^

Imaging modalities offer valuable information on structural changes in knee OA, enabling accurate diagnosis and clinical decision-making. Radiographic assessments are the most widely used and cost-effective imaging modality for depicting bony changes in knee OA, such as osteophyte formation, joint space narrowing, subchondral sclerosis, and subchondral cysts, which are widely applied radiographic criteria for establishing structural OA.^35^ However, radiography is unsuitable for early-stage OA diagnosis because of its inability to detect pathologic features before hallmark structural changes, such as osteophyte formation, occur.^34,37^ Magnetic resonance imaging (MRI) provides comprehensive visualization of pathologic changes, enabling the detection of early-stage OA structural alterations. However, its clinical application is hindered by the lack of reliable contrast agents to differentiate cartilage from surrounding synovial fluid, as well as the extended acquisition time (often exceeding 40 min) required to generate diagnostic-quality images.^35–37^ Computed tomography (CT) is not as commonly employed as radiography or MRI in clinical practice because of its inherently poor soft-tissue contrast; nevertheless, it remains a valuable tool for evaluating facet joint involvement in OA. It provides detailed visualization of cortical bone integrity and soft-tissue calcifications, thereby offering unique diagnostic insights into structural changes associated with the disease.^38^ Ultrasound imaging has emerged as a valuable modality for visualizing joint effusions, osteophytes, and other OA-related features. Its portability and cost-effectiveness have driven its widespread adoption for OA diagnosis in European and US centers. However, its utility in detecting joint space narrowing is less precise than that of MRI, and its detection depth is constrained by the operator’s expertise.^37,39^ Optical imaging, which provides quantitative information with high spatial resolution for structural OA diagnosis, faces significant limitations because of its shallow penetration depth, which is caused primarily by significant optical scattering within tissues.^37^ Photoacoustic imaging (PAI), an emerging optical imaging modality, leverages rich contrast, high spatial resolution, and deep penetration depth, thereby overcoming the limitations inherent in conventional optical imaging techniques and providing novel insights for OA diagnosis.^40,41^

## Osteoarthritis pathogenesis

Emerging evidence suggests that OA pathogenesis arises from maladaptive crosstalk between sustained joint inflammation and intracellular organelle dysfunction. Proinflammatory cytokines, notably IL-1β, IL-6, and TNF-α, initiate inflammatory cascades in synovial tissue and chondrocytes. Concurrently, disturbances in mitochondrial bioenergetics, ER stress, and lysosomal destabilization within chondrocytes amplify oxidative stress and trigger apoptotic pathways. This synergy creates a feedforward loop that accelerates extracellular matrix degradation, impairs cartilage homeostasis, and promotes subchondral bone remodeling. The convergence of inflammatory signaling and organelle failure likely drives the transition from early chondrocyte senescence to irreversible tissue destruction, highlighting the potential of dual-targeting strategies to halt OA progression.

### Role of proinflammatory cytokines in osteoarthritis

**Fig. 1:**
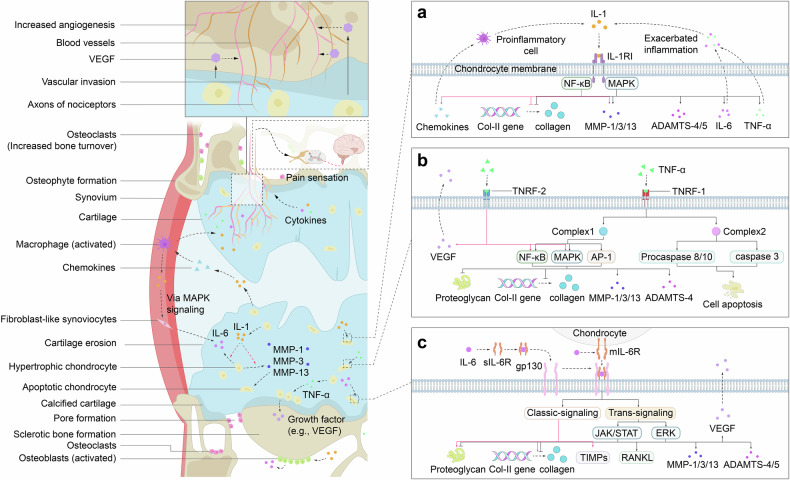
Schematic representation of the inflammatory signaling pathways and their impact on joint tissues in OA. The left panel illustrates the pathological changes within the joint, emphasizing the role of inflammatory cells and the degradation of the extracellular matrix. The right side delves into the molecular mechanisms underlying these changes, revealing the pivotal role of IL-1, TNF-α, and IL-6 in modulating the activity of key signaling pathways that drive ECM degradation and inflammation. **a** IL-1 signaling role in osteoarthritis progression. **b** TNF-α signaling role in osteoarthritis progression. **c** IL-6 signaling role in osteoarthritis progression. These findings underscore the complex feedback loops involving these cytokines and their downstream effectors, which contribute to the chronic inflammation and tissue remodeling characteristic of OA. The figure also incorporates the vascular aspects of OA, highlighting the role of VEGF in angiogenesis and its influence on chondrocyte survival and function. This schematic provides a visual summary of the current understanding of OA pathogenesis, emphasizing the multifactorial nature of the disease and the potential therapeutic targets within the inflammatory signaling cascades

Proinflammatory cytokines play crucial roles in OA pathogenesis, promoting a self-sustaining inflammatory environment within the knee joint. These cytokines perpetuate a vicious cycle through their own signaling pathways, intensifying pain sensation and disrupting joint metabolic balance. They enhance osteoclast activity, leading to bone resorption, and promote the secretion of catabolic enzymes and chondrocyte apoptosis,^42^ all of which contribute to detrimental alterations in joint morphology. Clinical trials have revealed elevated levels of IL-1β, IL-6, and TNF-α in the synovial fluid, synovial membrane, subchondral bone, and cartilage of patients with OA,^43,44^ supporting their role as key contributors to catabolic processes in OA pathogenesis (Fig. 1). A comprehensive understanding of their roles offers promising avenues for developing targeted therapies that may surpass the effectiveness of traditional anti-inflammatory treatments.

#### IL-1

The IL-1 family comprises IL-1α, IL-1β, and the IL-1 receptor antagonist (IL-1Ra), with IL-1α and IL-1β acting as agonists. Both IL-1α and IL-1β are critical proinflammatory cytokines involved in OA etiology, causing inflammation and ECM degradation in articular cartilage, as evidenced in clinical trials,^44–46^ ultimately leading to cartilage destruction. These cytokines are synthesized primarily as precursor peptides, pro-IL-1α and pro-IL-1β, in monocytes and macrophages. Pro-IL-1α is biologically active and can be cleaved by the cytosolic cysteine protease calpain to generate mature IL-1α, which has a greater binding affinity to IL-1 receptors.^47,48^ Interestingly, IL-1α is rarely detected in healthy human tissues but is substantially elevated in disease states, such as OA, suggesting that it may be released from lysed cells as an alarmin signal of cellular damage or necrosis.^48–50^ In contrast, pro-IL-1β is biologically inactive and requires cleavage by caspase-1 (also known as ICE) to become active IL-1β.^48,49,51^ IL-1β is produced by various cell types in the knee joint, including chondrocytes, mononuclear cells, osteoblasts, activated macrophages, and synovial tissues.^44–46^ It is found at elevated levels in the synovial fluid, synovial membrane, cartilage, and subchondral tissue of OA patients.^44,52^ Both IL-1α and IL-1β mediate inflammation by binding to type I IL-1 receptor I (IL-1RI), a membrane protein expressed in various cells, such as chondrocytes, synovial fibroblasts, osteoblasts, osteoclasts, and macrophages.^53–55^ IL-1RI contains three extracellular immunoglobulin-like domains and an intracellular Toll/IL-1R (TIR) domain.^48,56^ The interaction between IL-1 and IL-1RI activates NF-κB, leading to inflammatory responses.^50,56,57^

Additionally, IL-1 promotes the secretion of proinflammatory cytokines and mediators, including IL-6, TNF-α, nitric oxide (NO), cyclooxygenase-2 (COX-2), and PGE_2_.^58^ It also enhances the expression of matrix-degrading enzymes,^59^ particularly matrix metalloproteinases (MMPs) and disintegrin-like and metalloproteinase with thrombospondin motifs (ADAMTS), which mediate the degradation of ECM proteins, such as MMP-1, MMP-3, MMP-13, ADAMTS-4, and ADAMTS-5.^44,45,53,60^ IL-1 also impairs proteoglycan aggrecan and type II collagen synthesis by suppressing SRY-box transcription factor 9 (SOX-9),^60^ a critical gene whose protein product directs the transcription of genes encoding type II collagen and the proteoglycan aggrecan via the MAPK signaling pathway,^61^ further aggravating the inflammatory microenvironment initiated by IL-1.^44,53^

Recent studies have highlighted the critical role of IL-1 in activating the NF-κB signaling pathway, which exacerbates OA progression. NF-κB activation induces the expression of MMPs (MMP-1, MMP-2, MMP-3, MMP-7, MMP-8, MMP-9, and MMP-13) and ADAMTS family members (ADAMTS-4 and ADAMTS-5), as well as inflammatory mediators, including COX-2, PGE_2_, NO, inducible nitric oxide synthase (iNOS), and hypoxia-inducible factor-2α, all of which have been validated both in vitro (e.g., chondrocyte culture) and in vivo (e.g., murine OA models), and further enhances MMP expression.^53,62,63^ Additionally, NF-κB activation increases the secretion of cytokines and chemokines such as TNF-α, IL-1β, IL-8, monocyte chemoattractant protein-1 (MCP-1/CCL2), CCL5, macrophage inflammatory protein-1α (MIP-1α), and receptor activator of NF-κB ligand (RANKL).^53,63^ These chemokines recruit inflammatory cells, such as activated macrophages, which are a major source of IL-1β in the synovium, thereby contributing to a vicious cycle of IL-1-mediated catabolic events that accelerate OA progression (Fig. 1a).^53^

This IL-1-mediated signaling is subject to reversible regulation by the naturally occurring inhibitors IL-1Ra and IL-1RII. IL-1Ra, which is synthesized in the Golgi apparatus, is primarily secreted from the same cells that release IL-1β.^57^ Similar to IL-1α and IL-1β, IL-1Ra competitively binds to the same site on IL-1RI, exhibiting near-equal avidity, thus inactivating IL-1 signaling.^44,49,53,64^ Complete inhibition of IL-1 signaling requires a 100–1000-fold molar excess of IL-1Ra to generate IL-1, as even minimal engagement of IL-1 with IL-1RI is sufficient to initiate cellular inflammatory responses.^48,50^ IL-1RII, a member of the IL-1 receptor family, shares structural similarities with IL-1RI but lacks the TIR domain, making it a decoy receptor that does not initiate signal transduction.^48^ Additionally, IL-1RII exists in both membrane-bound and soluble forms, both of which exhibit a greater affinity for IL-1β than for IL-1α or IL-1Ra, which bind almost irreversibly to IL-1β.^48,56^

#### TNF-α

TNF-α is a pivotal proinflammatory cytokine that plays a central role in driving numerous inflammatory responses. Elevated TNF-α secretion, in combination with IL-1β, contributes to deleterious morphological changes in joints during OA progression by modulating gene expression, apoptosis, differentiation, proliferation, and inflammatory pathways.^53,65,66^ TNF-α is initially synthesized as a transmembrane form (tmTNF-α) by the same cell types and tissues that produce IL-1β, including chondrocytes, mononuclear cells, osteoblasts, and synovial tissues.^44,65^ This tmTNF-α, a stable homotrimeric type II transmembrane protein, can be cleaved by the metalloprotease TNF-α-converting enzyme (TACE) into its soluble form (sTNF-α), which is then released into the extracellular environment.^65^

TNF-α exerts its effects by binding to two cognate receptors, tumor necrosis factor receptor 1 (TNFR-1) and TNFR-2 (Fig. 1b). Recent studies indicate that TNFR-1 is primarily responsible for TNF-α-mediated signaling, as it can interact with both tmTNF-α and sTNF-α and is expressed in almost all cell types. In contrast, TNFR-2 preferentially binds to tmTNF-α and is expressed in a more limited subset of cells.^53,65^ These two receptors initiate distinct signal transduction pathways, largely due to differences in their intracellular domains. TNFR-1 contains a death domain (DD) that is absent in TNFR-2.^65^ Under resting conditions, TNFR-1 remains inactive as the silencer of the death domain (SODD) binds to the DD.^67^ Upon TNF-α binding, SODD dissociates from the DD, leading to TNFR-1 activation, which then recruits TNF receptor-associated death domain (TRADD) and other adapter proteins, such as receptor-interacting protein-1, TNFR-associated factor 2 (TRAF2), cellular inhibitor of apoptosis proteins 1 and 2 (cIAP1, cIAP2), and the linear ubiquitin chain assembly complex, to form complex I, which activates the NF-κB signaling pathway.^66,68^ Additionally, the MAPK signaling pathway is activated via TRAF2, leading to the nuclear translocation of activated JNK, which subsequently induces transcription factors such as activator protein 1 (AP-1).^69,70^ These combined pathways drive the degradation of the proteoglycan aggrecan, inhibit type II collagen expression, and upregulate MMP and ADAMTS secretion, as demonstrated both in vitro and in vivo.^44,53^ Under certain circumstances, the failure of complex I to activate the NF-κB pathway results in the inhibition of cFLIP, an NF-κB-induced protein that prevents the recruitment and activation of pro-caspase 8, protecting cells from apoptosis.^71^ Instead, the components of complex I (TRADD-RIP1-TRAF2) dissociate and bind to FAS-associated death domain proteins, forming complex II in the cytosol. Complex II recruits and activates pro-caspase 8, which activates caspase 3 to induce chondrocyte apoptosis.^66,67,70^ This disruption of the ECM repair process leads to a failure to maintain cartilage homeostasis, contributing to OA pathogenesis.^72^

The signaling mechanism of TNFR-2 is less well understood, but it is believed to support cell activation, migration, and proliferation.^68^ The binding of tmTNF-α to TNFR-2 facilitates the recruitment of TRAF2, TRAF1, cIAP1, and cIAP2, which assemble to activate both the NF-κB and MAPK signaling pathways. These pathways, as previously described, are implicated in driving inflammatory responses. Interestingly, studies by Takahito et al. revealed that ablation of TNFR-2 in TNF∆^ARE^ mice exacerbated joint inflammation, suggesting that TNFR-2 may act as a crucial anti-inflammatory mediator in OA progression.^73^

Healthy cartilage is an avascular tissue with low oxygen tension (1–5%), supporting the relatively slow metabolic activity of chondrocytes. However, as OA progresses, the microvasculature from the subchondral bone invades the calcified cartilage, increasing oxygen tension and promoting chondrocyte apoptosis.^74^ Angiogenesis, largely driven by chondrocyte-produced vascular endothelial growth factor, is further stimulated by TNF-α.^74,75^ Additionally, experimental studies in OA chondrocytes have demonstrated that TNF-α enhances the synthesis of various chemokines and inflammatory mediators, including IL-8, CCL5 (also known as RANTES), iNOS, COX-2, and PGE_2_,^53,65^ all of which exacerbate the inflammatory state within the joint. In conjunction with IL-1β-induced inflammatory responses, this further amplifies the production of TNF-α, creating a positive feedback loop that accelerates OA progression.

#### IL-6

IL-6 has long been recognized as a proinflammatory cytokine that plays a pivotal role in the catabolic processes of OA progression. However, emerging evidence suggests that IL-6 has a complex biological function in OA pathogenesis, contributing to both protective and detrimental effects. IL-6 is produced primarily by chondrocytes, osteoblasts, synovial fibroblasts, and the infrapatellar fat pad in OA.^76^ Notably, the infrapatellar fat pad is a major source of adipose tissue within the knee joint, implicating obesity as a significant risk factor for IL-6-driven OA.^77^

IL-6 exerts its effects by binding to two distinct forms of nonsignaling IL-6 receptor (IL-6R): the membrane-bound IL-6 receptor (mIL-6R) and the soluble IL-6 receptor (sIL-6R) (Fig. 1c). sIL-6R results either from the cleavage of mIL-6R by metalloproteases, including ADAM10 and ADAM17, or, to a lesser extent, from alternative splicing of the same mRNA encoding IL-6R.^78,79^ The binding of IL-6 to IL-6R results in the formation of a complex that subsequently associates with glycoprotein 130 (gp130), a ubiquitously expressed transmembrane protein responsible for IL-6 signal transduction.^77,80^ The signaling pathway involving mIL-6R is termed the “classic-signaling pathway,” whereas the pathway involving sIL-6R is known as the “trans-signaling pathway.”^81–83^ Both pathways initiate similar downstream signaling cascades via gp130,^84^ but the trans-signaling pathway is considered dominant, as IL-6 preferentially binds to sIL-6R. The resulting complex is capable of inducing IL-6 signaling in a variety of cells expressing gp130 via the circulatory system, whereas mIL-6R is expressed on a limited range of cell types, including macrophages, osteocytes, chondrocytes, monocytes, leukocytes, and hepatocytes.^53,85,86^

Additionally, a soluble form of gp130 (sgp130) is observed in the bloodstream. Sgp130 functions as an inhibitor of the IL-6-induced trans-signaling pathway by binding to IL-6-sIL-6R complexes with higher affinity than to IL-6-mIL-6R complexes.^78,87^ Upon binding, receptor homodimerization brings Janus kinases (JAKs), including JAK1, JAK2, and tyrosine kinase 2 (TYK2), into proximity, resulting in their activation. This activation phosphorylates tyrosine residues in the intracellular domains of gp130, triggering two main gp130-mediated signaling pathways: the signal transducer and activator of transcription (STAT) pathway, which facilitates the translocation of transcription factors such as STAT1, STAT3, and STAT5 into the nucleus to regulate target gene expression, and the ERK pathway, which is activated by the phosphorylation of tyrosine 759.^77,80,82,85,88^ Both signaling pathways are tightly regulated by suppressors of cytokine signaling (SOCS), which are downstream target genes of STAT3. Specifically, SOCS-1 and SOCS-3 are transcribed in response to JAK/STAT activation. SOCS-1 inhibits JAK/STAT signaling by binding to JAKs, while SOCS-3 associates with tyrosine 759 to terminate ERK signaling.^78,82,88^

Classic-signaling and trans-signaling pathways involving IL-6 could play opposing roles in OA pathogenesis. The classic-signaling pathway is typically associated with protective effects, such as increasing proteoglycan aggrecan synthesis in chondrocytes and promoting the production of tissue inhibitors of metalloproteinases, which counteract the effects of MMPs.^86,88^ In contrast, the trans-signaling pathway is thought to drive catabolic events in human OA chondrocytes, as evidenced by the observed inhibition of proteoglycan synthesis and the upregulation of cartilage-degrading enzymes.^78,89^ However, recent studies suggest that both pathways can induce opposite effects under different conditions,^86,90^ although the underlying mechanisms for this discrepancy remain unclear. The balance between the classic and trans-signaling pathways in chondrocytes is possibly influenced by mIL-6R expression, which can be upregulated by IL-1β or downregulated by transforming growth factor-β (TGF-β). Disruption of this balance may partially explain the shift between anabolic and catabolic events mediated by IL-6.^86^

Overall, IL-6 upregulates the expression of MMPs (MMP-1, MMP-3, and MMP-13) and ADAMTS family members (ADAMTS-4 and ADAMTS-5) in chondrocytes via the JAK/STAT and ERK signaling pathways.^76,90^ Furthermore, IL-6 stimulates the expression of RANKL via JAK/STAT signaling in synovial fibroblasts, disrupting the homeostasis between bone formation and resorption orchestrated by osteoblasts and osteoclasts, thus facilitating osteoclastogenesis and contributing to bone turnover. This leads to deleterious morphological changes in subchondral bone.^78,82,86^ IL-6 released by synovial fibroblasts acts synergistically with TNF-α to increase vascular endothelial growth factor production, which elevates oxygen tension, induces interstitial edema, and increases tissue pressure by increasing vascular permeability. These effects result in chondrocyte apoptosis and tissue damage.^78,82,90^ Furthermore, SOCS-3, which is induced by IL-6, inhibits chondrogenesis by reducing the levels of insulin-like growth factor-1 (IGF-1), an essential mediator of ECM, collagen II, and proteoglycan synthesis, as well as the differentiation of chondrogenic progenitor cells. Moreover, SOCS-3 also dampens IL-6 signaling, highlighting the paradoxical role of IL-6 in OA progression.^86,88^

### Role of chondrocyte organelles in osteoarthritis

Chondrocytes, the only cellular component of cartilage, play crucial roles in maintaining the structural integrity and function of articular cartilage. The preservation of normal cartilage morphology relies on a dynamic balance of chondrocyte function, wherein the health and activity of chondrocyte organelles are essential. Disruptions in organelle homeostasis during OA pathology can lead to detrimental changes in chondrocyte differentiation and even apoptosis, which further exacerbate cartilage degeneration. A deeper understanding of the roles of chondrocyte organelles in OA progression offers valuable insights for identifying novel therapeutic targets and strategies aimed at preserving chondrocyte functionality.

#### Mitochondria

Mitochondria play a central role in cellular metabolism by producing the majority of adenosine triphosphate (ATP), which is essential for various physiological processes across nearly all human tissues. Mitochondrial dysfunction has been closely linked to several age-related diseases, including OA. Recent studies suggest that an imbalance between mitochondrial biogenesis and the clearance of damaged mitochondria contributes to OA onset and progression in chondrocytes.^91,92^

**Fig. 2:**
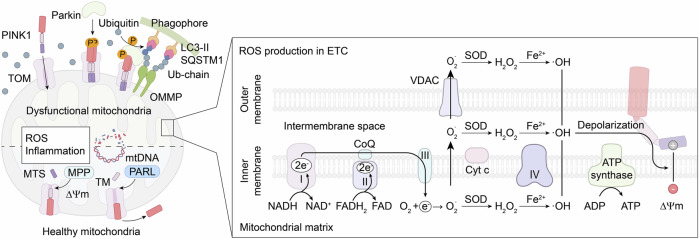
Role of mitochondria in the progression of OA. In healthy mitochondria, the ETC chain in the IMM provides sufficient ATP production to maintain chondrocyte homeostasis, and complexes I-III of the ETC chain have the capacity for ROS generation. Under normal circumstances, PINK1 is transported by TOM through the OMM and IMM, where it is retained in the central matrix with the assistance of the ΔΨm. In the matrix, PINK1’s MTS and TM domains are cleaved by MPP and PARL, leaving an unstable PINK1 remnant that is subsequently degraded via the ubiquitin–proteasome system. Consequently, Parkin remains in an inactive state, preventing mitophagy from occurring. Mitochondrial dysfunction, resulting from mtDNA damage, inflammasome activation, or ROS attack, leads to a depolarized ΔΨm, which prevents PINK1 from remaining in the matrix. The PINK1 accumulated on the OMM undergoes transphosphorylation, which activates Parkin to ubiquitinate OMM proteins, which bind to the phagophore via LC3-II localized on the phagophore membrane directly or through SQSTM1, which possesses an LC3-interacting region to bind to the phagophore indirectly. The phagophore subsequently matures into an autophagosome, which fuses with the lysosome to degrade damaged mitochondria. Appropriate mitophagy helps eliminate excessive ROS and maintain chondrocyte homeostasis, but excessive mitophagy can lead to chondrocyte apoptosis due to an insufficient energy supply. ETC electron transport chain, IMM inner mitochondrial membrane, ATP adenosine triphosphate, ROS reactive oxygen species, PINK1 PTEN-induced putative kinase 1, TOM translocases of the outer membrane, OMM outer mitochondrial membrane, OMMP outer mitochondrial membrane proteins, ΔΨm mitochondrial membrane potential, MPP mitochondrial processing peptidase, PARL presenilin-associated rhomboid-like protease, mtDNA mitochondrial DNA, LC3-II autophagic adapter protein light chain 3-II, SQSTM1 p62/Sequestosome 1

Mitochondria are composed of four main structural components: the central matrix, the inner mitochondrial membrane (IMM), the outer mitochondrial membrane (OMM), and the intermembrane space.^93^ The electron transport chain (ETC), located within the IMM, plays a pivotal role in ATP generation and maintaining the mitochondrial membrane potential (ΔΨm).^93,94^ The ETC, composed of complexes I-IV, ubiquinone, and cytochrome c, facilitates electron transfer, coupled with proton (H^+^) pumping across the membrane by complexes I, III, and IV. This proton gradient provides the electrochemical energy required for ATP synthase (Complex V) to convert ADP into ATP.^93,95^ However, this process generates byproducts: electrons from complexes I and II prematurely react with molecular oxygen (O_2_), potentially forming superoxide anions (O_2_^−^). These anions are detoxified by superoxide dismutase into hydrogen peroxide (H_2_O_2_), which, in the presence of metal ions such as Fe^2+^ and Cu^2+^, can generate highly reactive hydroxyl radicals (·OH). Moreover, superoxide anions can be translocated to the mitochondrial matrix through voltage-dependent anion channels (VDACs), leading to further ·OH formation and impacting the ΔΨm (Fig. 2).^92,93,95^ These reactive oxygen species (ROS) are implicated in the pathogenesis of OA, highlighting the delicate balance between energy production and oxidative stress. Although mitochondria are crucial for energy production, some studies suggest that chondrocytes predominantly rely on glycolysis for ATP synthesis because of their low-oxygen environment and lack of innervation, with only ~25% of ATP generated through mitochondrial oxidative phosphorylation.^92,95^ Nonetheless, a decrease in ATP production is observed in OA chondrocytes, which is attributed to reduced oxidative phosphorylation and a decrease in the number of mitochondria. These findings suggest that mitochondria are pivotal in OA progression despite the increased glycolytic activity in these cells.^92^

Mitochondrial biogenesis, the process by which mitochondria divide to generate new organelles, is regulated primarily by peroxisome proliferator-activated receptor γ coactivator 1α (PGC-1α).^96^ PGC-1α activates downstream transcription factors, including the nuclear respiratory factors NRF1 and NRF2, which promote mitochondrial transcription factor A (TFAM) expression. This, in turn, initiates mitochondrial DNA (mtDNA) replication and the production of new mitochondria.^96,97^ AMP-activated protein kinase (AMPK) modulates PGC-1α activity either through direct phosphorylation or by activating Silent Information Regulator 1 (SIRT1), a mitochondrial histone deacetylase that also influences PGC-1α activity.^97,98^ Studies have shown reduced mitochondrial biogenesis and an accumulation of dysfunctional mitochondria in OA chondrocytes. This imbalance leads to excessive ROS production, damages mitochondrial DNA and impairs ATP generation, contributing to chondrocyte apoptosis and cartilage degradation.^92,99^

In recent years, mitophagy has emerged as a potential therapeutic target for OA and other bone diseases. Mitophagy is the selective removal of damaged or dysfunctional mitochondria to protect cells from apoptosis.^100^ This process can occur via two main pathways: the Parkin RBR E3 ubiquitin-protein ligase (PRKN)-dependent pathway and the PRKN-independent pathway, with the former being the most well studied.^91,93,100,101^ PINK1 is a serine/threonine (Ser/Thr) kinase comprising an N-terminal mitochondrial targeting sequence (MTS), followed by an α-helical membrane (TM) segment and a Ser/Thr kinase domain, whereas parkin is an E3-ubiquitin ligase with an N-terminus termed the UBL domain.^93,102^ Under normal physiological circumstances, PINK1 is imported into mitochondria, where translocases of the outer membrane (TOM) carry the MTS of PINK1 through the OMM and IMM of mitochondria, allowing the MTS to be exposed to the central matrix with the help of the ΔΨm.^93^ The MTS is then cleaved by mitochondrial processing peptidase in the central matrix, and the TM segment is cleaved by presenilin-associated rhomboid-like protease (PARL) in the IMM. This process results in an unstable PINK1 remnant, which is released from the mitochondria to the cytosol for rapid degradation by the ubiquitin protease system.^93,102^ Moreover, the UBL domain of parkin inhibits its own E3-ubiquitin ligase activity at the N-terminus, resulting in inhibited mitophagy in normal cells.^93^ However, under pathological conditions, the mitochondria may depolarize due to the negative effects of proinflammatory mediators, mtDNA damage, or reactive oxygen species (ROS) attack, as experimentally confirmed in human OA chondrocytes.^95,103^ These events inhibit the translocation of the MTS of PINK1 into the central matrix, leading to the cessation of PINK1 degradation.^93,102^ Instead, accumulated PINK1 binds with TOM on the OMM surface as an integral molecule that undergoes transautophosphorylation to form complexes concomitant with high Parkin kinase activity, which recruits cytosolic Parkin to malfunctioning mitochondria.^102,104^

Once recruited, Parkin is activated by PINK1 phosphorylation at Ser65 in its UBL domain, which relieves its inhibitory effect on E3 ligase activity, allowing Parkin to phosphorylate ubiquitin (ph-Ub) and poly-Ub chains that bind to a plethora of OMM proteins, including mitofusin 1 (MFN1), MFN2, mitochondrial Rho-GTPase 1 (Miro1), and VDAC1. The ubiquitinated OMM proteins are capable of binding with phagophores via the autophagic adapter protein light chain 3-II (LC3-II) localized on the phagophore membrane directly or through p62/Sequestosome 1 (SQSTM1), which contains an LC3 interaction region that binds to phagophores indirectly.^91,102^ The phagophores subsequently mature into autophagosomes, which fuse with lysosomes to degrade damaged mitochondria (Fig. 2).^105,106^

#### Lysosome

Lysosomes, single-membrane organelles, are essential for degrading cellular waste and dysfunctional organelles through various hydrolytic enzymes within an acidic lumen and are maintained by the multisubunit V-ATPase complex.^107,108^ Autophagy occurs in three main forms, classified by substrate delivery to lysosomes: microautophagy, macroautophagy, and chaperone-mediated autophagy.^107,109^ Microautophagy involves the direct engulfment of small cytosolic particles into lysosomes for degradation, whereas macroautophagy is initiated by the formation of phagophores, which capture larger cytosolic material or damaged organelles. Phagophores mature into autophagosomes, which fuse with lysosomes to degrade their cargo.^107,109,110^ CMA is a selective autophagic process that recognizes specific protein motifs, facilitating their translocation to lysosomes for degradation via the heat shock cognate protein of 70 kDa (HSC70).^111,112^

Lysosomal cathepsins, a family of ~12 proteases, play a pivotal role in breaking down macromolecules by cleaving peptide bonds within the lysosomal matrix.^107^ Notably, studies indicate that chondrocytes internalize hydroxyapatite, a primary crystal contributing to cartilage calcification in OA.^113,114^ Excessive hydroxyapatite is transferred to lysosomes for dissolution through acidification,^115,116^ leading to a compromised acidic milieu within the lysosome that dramatically affects soluble lysosomal hydrolases, including acid sphingomyelinase (ASM), which modulates the balance of lysosomal lipid composition (particularly sphingomyelin), cathepsins responsible for dissolved hydroxyapatite matrix degradation, and lysosome-associated membrane proteins (LAMPs), which are critical for maintaining lysosomal morphology. This results in increased oxidative stress, accumulated dysfunctional mitochondria, and lysosomal membrane permeabilization (LMP).^107,117^

**Fig. 3:**
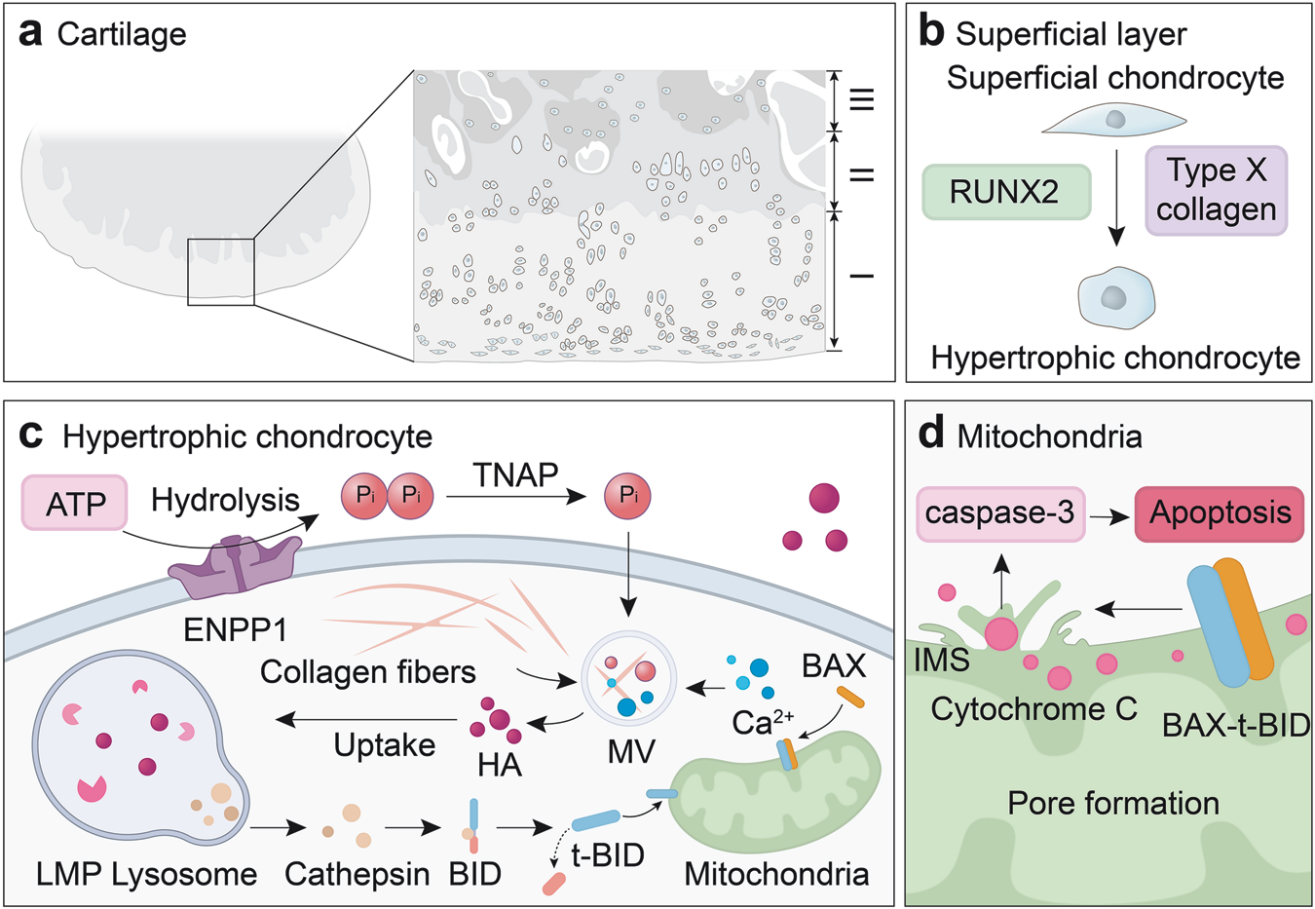
Mechanisms of lysosome-induced chondrocyte apoptosis. **a** Histological stratification of cartilage microstructure. **b** RUNX2-driven hypertrophic transition of superficial chondrocytes with type X collagen secretion as a hypertrophy biomarker. **c** Hydroxyapatites, derived from both chondrocyte uptake and hypertrophic chondrocyte synthesis, are transported to lysosomes for processing. This process may lead to LMP, which causes the release of cathepsins into the cytosol. The cathepsins cleave the proapoptotic protein BID into its active form, t-BID, which inserts into the OMM and recruits BAX. **d** The t-BID-BAX complex forms pores in the OMM, resulting in the leakage of cytochrome c from the IMS into the cytosol. Cytochrome c then activates caspase-3, triggering chondrocyte apoptosis. Abbreviations: I superficial layer, II transition layer, III deep layer, TNAP tissue-nonspecific alkaline phosphatase, MV matrix vesicle, HA hydroxyapatite, LMP lysosomal membrane permeabilization, OMM outer mitochondrial membrane, IMS intermembrane space

LMP triggers the release of cathepsins, particularly cathepsins B and D, into the cytosol, where they cleave the proapoptotic protein BID into its active form, t-BID,^118,119^ which translocates and inserts into the OMM, where it facilitates the recruitment of BAX, another cytosolic proapoptotic protein.^120^ BAX, upon binding to t-BID, oligomerizes to form pores in the OMM.^121,122^ The mitochondrial outer membrane permeabilization (MOMP) eventuates the release of cytochrome C, a protein that originally mediates electron transfer between complexes III and IV of the ETC and detoxifies ROS in the intermembrane space. However, once released into the cytosol, cytochrome C initiates chondrocyte apoptosis and exacerbates catabolic processes in OA by activating caspase 3 (Fig. 3).^117^

Targeting hydroxyapatite removal may offer a potential therapeutic strategy for mitigating LMP-induced chondrocyte apoptosis in OA. Understanding the mechanisms underlying hydroxyapatite formation is imperative. In healthy cartilage, chondrocytes in the superficial and middle layers remain uncalcified, while mineralizing hypertrophic chondrocytes, characterized by high expression of Runt-related transcription factor 2 (RUNX2) and type X collagen, are localized in the deep cartilage layers.^123^ RUNX2 regulates chondrocyte differentiation into mineralizing hypertrophic cells, with type X collagen serving as a hypertrophy marker.^123–125^ Pathological calcification occurs when uncalcified chondrocytes in the superficial layer aberrantly differentiate into mineralizing hypertrophic cells.^126^

The enzyme ectonucleotide pyrophosphatase/phosphodiesterase family member 1, located on hypertrophic chondrocyte membranes, converts ATP into inorganic pyrophosphate,^127,128^ which is hydrolyzed by tissue-nonspecific alkaline phosphatase into inorganic phosphate (P_i_).^124,126,127^ The sodium-dependent phosphate transporters PiT-1 and PiT-2 transport excess P_i_ into matrix vesicles (MVs) secreted by hypertrophic chondrocytes.^124,129^ Within MVs, P_i_ interacts with calcium (Ca²⁺) internalized via Annexin V to form an amorphous calcium phosphate precursor (ACP).^130^ Upon interaction with collagen fibrils, ACP is converted into hydroxyapatite and released into the cytosol.^123^ Additionally, mitochondria are integral in maintaining Ca²⁺ and P_i_ homeostasis.^127,131^ Dysfunctional mitochondria contribute to the formation of ACPs, which are then delivered to lysosomes via mitophagy.^123^ However, conflicting clinical data suggest that mitophagy may play a protective role in maintaining chondrocyte survival.^93,100,101^ Further research is needed to elucidate the precise mechanisms linking mitophagy and cartilage calcification.

#### Endoplasmic reticulum

**Fig. 4:**
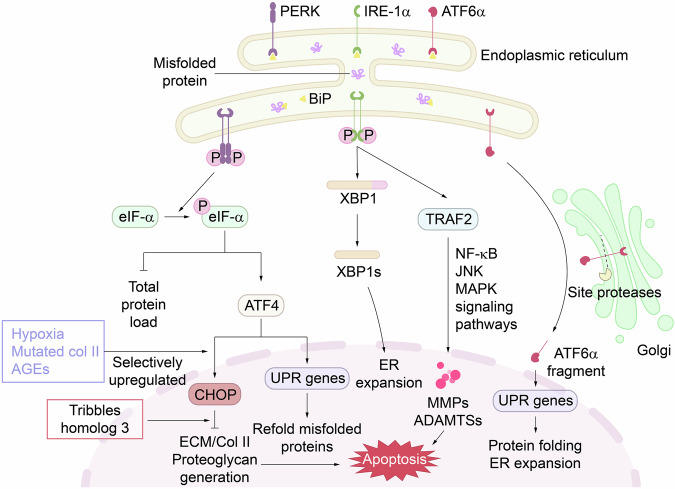
Illustration of the ER stress response and its implications in OA. The diagram depicts the activation of the UPR due to the accumulation of misfolded proteins within the ER. Key ER stress sensors, including PERK, IRE1α, and ATF6α, are activated upon dissociation from the chaperone BiP. PERK phosphorylates eIF2α to attenuate protein synthesis, whereas IRE1α splices XBP1 mRNA to generate the active transcription factor XBP1s, which promotes the expression of chaperones and proteins involved in ER expansion. Upon cleavage, ATF6α translocates to the nucleus to activate UPR target genes that aid in protein folding and ER homeostasis. The figure also highlights the role of hypoxia, mutated collagen II, and AGEs in promoting ER stress. Prolonged ER stress can lead to the activation of proapoptotic pathways, including the CHOP pathway, and the production of matrix-degrading enzymes such as MMPs and ADAMTSs, contributing to cartilage degradation in OA. UPR unfolded protein response, PERK protein kinase-RNA-like endoplasmic reticulum kinase, IRE1α inositol-requiring enzyme 1 alpha, ATF6α activating transcription factor 6α, BiP glucose-regulated protein 78, eIF2α eukaryotic translation initiation factor 2A, ATF4 activating transcription factor 4, CHOP C/EBP homologous protein, XBP1 X-box binding protein 1, XBP1s spliced X-box binding protein 1, TRAF2 TNFR-associated factor 2, AGEs advanced glycation end products

The ER is the largest membrane-bound organelle adjacent to the outer membrane of the eukaryotic cell nucleus. It is the major site of protein synthesis, lipid biogenesis, detoxification, and intracellular Ca²⁺ storage.^132^ In the early stages of OA, environmental insults or increased protein synthesis—stemming from the attempts of chondrocytes to repair damaged cartilage—often lead to the accumulation of misfolded proteins within the ER, resulting in ER stress that negatively affects cellular activity and viability (Fig. 4).^133^

Mild ER stress, characterized by limited accumulation of misfolded proteins, can be managed through the unfolded protein response (UPR). This protective mechanism aims to restore ER homeostasis by eliminating excess misfolded proteins via two key mechanisms: ER-associated degradation (ERAD) and autophagy (ER-phagy). ER-phagy, akin to mitophagy, involves the sequestration of dysfunctional organelles into autophagosomes, which subsequently fuse with lysosomes for degradation.^133,134^

In contrast, ERAD activation is mediated by three transmembrane ER proteins—protein kinase-RNA-like ER kinase (PERK), inositol-requiring enzyme 1 alpha (IRE1α), and activating transcription factor 6α (ATF6α)—which function as stress sensors.^133–135^ Under normal conditions, BiP (also known as glucose-regulated protein 78, GRP78) binds these sensors to keep them inactive in the absence of misfolded proteins. However, when misfolded proteins accumulate in the ER lumen, BiP dissociates from the sensors to bind the exposed hydrophobic regions of the misfolded proteins with higher affinity.^135^ The compensatory upregulation of BiP is consistently observed in both the cartilage and synovial fluid of OA patients, reflecting an adaptive effort to restore ER homeostasis under persistent stress conditions.^136–138^

BiP dissociation from PERK triggers PERK oligomerization and autophosphorylation, leading to the phosphorylation of eukaryotic translation initiation factor 2A (eIF2α). This inhibits cap-dependent translation to reduce the protein load in the ER.^133,134^ Phosphorylated eIF2α also promotes the translation of cap-independent mRNAs, including ATF4, which upregulates UPR target genes encoding chaperones to refold misfolded proteins. Additionally, ATF4 selectively activates C/EBP homologous protein (CHOP), a proapoptotic factor that triggers apoptosis if stress remains unresolved.^133,135^ Notably, CHOP expression is markedly upregulated in both the cartilage and synovial fluid of OA patients, indicating exacerbated CHOP-induced chondrocyte apoptosis in OA pathogenesis.^136–138^ SIRT1 has been shown to inhibit CHOP-induced apoptosis, making it a promising therapeutic target for mitigating ER stress.^139^

Following BiP dissociation, IRE1α dimerizes and undergoes autophosphorylation, acquiring endonuclease activity that splices X-box binding protein 1 (XBP1) mRNA, converting it into the functional transcription factor XBP1s. XBP1s upregulates protein chaperones, disulfide isomerases, and components of the protein translocation machinery, contributing to ER expansion. It also enhances the expression of ERAD components, supporting the recovery of ER homeostasis.^133–135^

Similarly, BiP dissociation from ATF6α enables its incorporation into a vesicle that migrates to the Golgi apparatus, where ATF6α is cleaved by site-1 (S1P) and site-2 proteases (S2P). The resulting active fragment translocates to the nucleus to activate UPR target genes involved in protein folding, secretion, ERAD, and ER expansion.^134,135^ Notably, ATF6α upregulation in OA chondrocytes augments XBP1s expression, as observed in OA cartilage biopsies, mediating adaptive responses that attenuate ER stress-induced chondrocyte apoptosis and ECM degradation.^136,140^ However, if ER stress persists, the UPR shifts toward a proapoptotic cascade through both CHOP-dependent and CHOP-independent pathways, culminating in chondrocyte apoptosis.^133,134^

Pathological ER stress has been implicated in OA progression. Factors such as hypoxia, mutated collagen II aggregates, and the accumulation of advanced glycation end products—an unavoidable byproduct of cellular senescence—contribute to ER stress in OA chondrocytes.^133,134,138^ Notably, ER stress upregulates tribbles homolog 3 (TRB3), a CHOP downstream proapoptotic protein that impairs the chondrocyte response to IGF-1, reducing ECM, collagen II, and proteoglycan production.^138^

Additionally, some studies suggest that IRE1α can activate the NF-κB, JNK, and MAPK signaling pathways through TRAF2, promoting the production of MMPs and ADAMTSs, which drive catabolic events in OA.^134,138^ Consistent with these findings, the expression of UPR sensors (ATF6α, PERK, and IRE1α) is markedly increased in OA cartilage compared with normal tissue, which is correlated with the degree of cartilage degradation.^138,141,142^ Thus, excessive ER stress plays a critical role in OA progression, highlighting the need for therapeutic strategies targeting ER stress pathways.

### Organelle dysfunction and inflammation in osteoarthritis

Emerging evidence implicates organelle dysfunction as a critical driver of inflammatory cascades in OA, with pattern recognition receptors (PRRs) upregulated in OA chondrocytes playing a central mechanistic role. These PRRs, encompassing four principal classes—NOD-like receptors, Toll-like receptors, retinoic acid-inducible gene-I-like receptors, and C-type lectin receptors—are activated by both exogenous ligands and endogenous damage-associated molecular patterns (DAMPs), including mtDNA, cardiolipin, ROS, and metabolites (e.g., ATP).^143–145^

Mitochondria, widely recognized as evolutionary remnants of ancestral Alphaproteobacteria (the precursors of modern gram-negative bacteria), exhibit molecular similarities to bacterial components.^144–146^ Under physiological conditions, the integrity of the mitochondrial double membrane prevents the release of these DAMPs, thereby preventing inflammatory activation. However, in OA, pathological insults such as proinflammatory cytokines (e.g., IL-1, IL-6, and TNF-α) or ROS induce mitochondrial dysfunction. This triggers BAX/BAK-mediated MOMP, followed by osmotic pressure elevation within the mitochondrial matrix that displaces the IMM into the cytosol, ultimately leading to IMM rupture and the release of DAMPs (e.g., mtDNA and ATP) into the extracellular space—a mechanism corroborated by the elevated levels of DAMPs and ATP detected in OA synovial fluid.^95,144^

Cytosolic mtDNA induces inflammatory responses through interactions with specific intracellular receptors. Specifically, mtDNA activates the NLRP3 inflammasome, a multiprotein complex containing caspase-1, thereby promoting the maturation of IL-1β.^144,145^ Additionally, mtDNA engages with TLR9 expressed on the ER, activating the downstream MAPK and NF-κB signaling pathways.^145^ Furthermore, cytosolic mtDNA, either alone or in complex with curvature-associated proteins such as TFAM or high mobility group box 1 (HMGB1), activates the cGAS-STING pathway, which orchestrates NF-κB signaling and exacerbates inflammatory responses in OA.^144,146^

While the causal relationship between proinflammatory cytokines and LMP remains ambiguous, evidence confirms that under OA conditions, the combined pathological effects of hydroxyapatite crystal deposition and ROS-enriched microenvironments directly induce LMP.^112,113^ This lysosomal destabilization enables the leakage of multiple cysteine cathepsins (B, L, V, S, X, and Z), which play both specialized and overlapping functional roles in NLRP3 inflammasome activation. Although the exact mechanisms remain unclear, potential mechanisms may involve cysteine cathepsins, K⁺ efflux, oxidative stress, and Ca²⁺/Na⁺ influx.^142^

ER stress induced by proinflammatory cytokines, oxidative stress, and hypoxia in chondrocytes drives inflammatory responses through the UPR, which is regulated by three primary signaling pathways: the PERK, IRE1α, and ATF6α pathways.^133,147,148^ Specifically, PERK activation leads to eIF2α phosphorylation, which reduces IκB synthesis and consequently activates NF-κB signaling.^149^ IRE1α activation triggers TRAF2-mediated IκB degradation, further activating NF-κB while also engaging in JNK signaling. ATF6α similarly promotes IκB degradation to increase NF-κB activity.^150^ Notably, cytoplasmic leakage of BiP directly activates NF-κB, orchestrating a multifaceted inflammatory response in OA.^150^

## Clinical manifestations of osteoarthritis

OA is characterized by structural joint degeneration, including osteophyte formation, cartilage defects, narrowed joint space, and subchondral bone remodeling. OA pain, one of the most prominent symptoms encouraging patients to seek medical intervention, often persists irrespective of these morphological alterations. Despite advances in OA treatment, current therapies for managing OA pain are largely palliative and unsatisfactory.^151^ None of the first-line agents, such as NSAIDs, opioids, hyaluronic acid hydrogels, or surgical procedures, have consistently demonstrated effective therapeutic outcomes.^41^ Over 75% of OA patients report the ongoing need for symptomatic relief.^151^

Morphological changes, including cartilage degradation, osteophyte formation, and periarticular tissue damage, have historically been regarded as the primary sources of pain in OA.^41^ However, imaging studies have revealed a relationship between the radiographic severity of OA and the intensity of pain experienced, suggesting that individual variations in pain perception may be influenced by genetic, environmental (e.g., obesity), psychological, and neurological factors. Given the poorly understood mechanisms underlying OA pain, the following section aims to systematically discuss the genesis and transmission of OA pain in the knee joint, offering insights into novel pain management strategies.

### Osteoarthritis pain genesis

The action potentials generated by peripheral stimuli at the knee joint are transmitted predominantly through a dense network of sensory afferent neurons, which can be classified into large-diameter myelinated Aβ fibers, medium-diameter myelinated Aδ fibers, and small-diameter unmyelinated C fibers on the basis of differences in conduction velocity and stimulus thresholds.^152^ Nociceptive pain signals are transmitted primarily by Aδ and C fibers, both of which are pseudounipolar neurons typically referred to as nociceptors.^153,154^ Owing to their larger diameters, Aδ-fibers conduct pain signals more rapidly and are mainly responsible for transmitting sharp pain, whereas C-fibers convey more diffuse, burning pain.^155,156^

The cell bodies of nociceptors are clustered in the dorsal root ganglion (DRG) adjacent to the spinal cord, where each nociceptor gives rise to two axons: one extending toward peripheral tissues—including the capsule, ligaments, meniscus, periosteum, subchondral bone, and synovium of the knee joint—and the other projecting toward the dorsal horn of the spinal cord.^154–159^ Nociceptor nerve endings in the periphery express transient receptor potential (TRP) channels, including TRPV1, TRPA1, TRPM3, and TRPM8.^160,161^ The activation of these channels by proinflammatory cytokines (IL-1 and TNF-α) and ROS transforms noxious stimuli into electrical signals by initiating Ca²⁺ influx.^162–165^ Following this transduction, voltage-gated sodium (Na_v_) channels, such as Na_v_1.1, Na_v_1.6, Na_v_1.7, Na_v_1.8, and Na_v_1.9, amplify these signals via Na^+^ influx, ultimately reaching the threshold for action potential generation.^157,166,167^

Nerve growth factor (NGF) also plays a vital role in peripheral pain generation. Both local and systemic administration of NGF induce robust, long-lasting mechanical and thermal hyperalgesia in rodents and human volunteers, underscoring its proalgesic properties.^168^ In OA joints, cytokines such as TNF-α and IL-1 stimulate NGF secretion by nonneural cells such as macrophages, mast cells, fibroblast-like synoviocytes, and keratinocytes. This leads to elevated NGF levels, potentially contributing to the aberrant growth of sensory neurons and sympathetic fibers in otherwise aneural cartilage.^169,170^ NGF exerts its effects through two receptors localized on peripheral nociceptors: high-affinity tyrosine receptor kinase A (TrkA) and low-affinity p75 neurotrophin receptor (p75^NTR^).^169,171^ NGF binding to TrkA activates three key intracellular signaling pathways: the mitogen-activated protein kinase (MAPK), phospholipase C-γ, and phosphoinositide 3-kinase pathways.^168,172^ These NGF–TrkA complexes, along with activated signaling intermediates, are transported via endosomes to DRG neuron cell bodies, where they promote sensory neuron sprouting and upregulate the expression of pain-related proteins such as TRPV1, Na_v_1.8, substance P (SP), and calcitonin gene-related peptide (CGRP).^168–170,173^ Additionally, p75^NTR^ enhances the sensitivity of TrkA to NGF by dimerizing with it, as p75^NTR^ itself lacks catalytic activity.^168,173^ This NGF–p75^NTR^ complex can mediate homeostatic responses in sensory neurons by inducing either apoptosis through c-Jun N-terminal kinase signaling or survival through the NF-κB pathway.^171,174,175^

The other end of Aδ-fiber axons terminates in laminae I and V of the dorsal horn of the spinal cord,^152,156,176^ where they transmit pain signals by directly synapsing with second-order neurons, such as spinothalamic and spinoreticular cells.^156,176^ The cell bodies of spinothalamic neurons are located primarily in laminae I and V, whereas spinoreticular neurons are found predominantly in laminae VII and VIII.^156,177,178^ In contrast, C-fiber axons terminate in laminae II, where they synapse with interneurons before transmitting pain signals to second-order neurons.^152,156^ Additionally, C-fibers are divided into peptidergic and nonpeptidergic fibers on the basis of their ability to produce SP and CGRP. Notably, bones are innervated by peptidergic but not nonpeptidergic C-fibers.^153,176^

Upon activation by noxious stimuli, the terminal endings of both types of nociceptors release neurotransmitters—including glutamate (Glu), SP, and CGRP—into the synaptic cleft to act on receptors of second-order neurons.^176,179^ In presynaptic terminals, Glu is packaged into vesicles via vesicular glutamate transporter 2 (VGLUT2), which facilitates its release.^180^ Once released, Glu binds to postsynaptic glutamate receptors, including metabotropic (mGluRs) and ionotropic (iGluRs) receptors located on second-order neurons.

Both SP and CGRP are synthesized in nociceptors and stored in dense-core vesicles under unstimulated conditions.^180–182^ Following neuronal depolarization, SP and CGRP are transported by fast axonal transport to the terminal endings of nociceptors. SP is released into the synaptic cleft to transmit noxious signals by binding with neurokinin 1 G-protein-coupled receptors (NK1Rs) on second-order neurons,^182,183^ whereas CGRP binds to a unique G protein-coupled receptor composed of three subunits: calcitonin-like receptor (CLR), receptor activity-modifying protein 1 (RAMP1), and receptor component protein (RCP).^184^

**Fig. 5:**
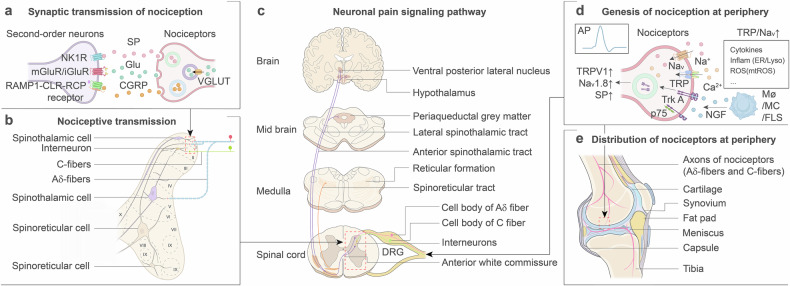
Overview of pain transmission in the knee joint. **a** Synaptic interface between primary afferent nociceptors and second-order neurons in the spinal dorsal horn. **b** Connectivity patterns and regional distribution of nociceptors and second-order neurons in the spinal cord. **c** Neuronal pain signaling pathway. **d** Magnified peripheral nociceptor terminals showing Nav and TRP channel cooperation with NGF to initiate pain signaling during noxious chemical and mechanical stimulation. **e** The distribution of nociceptors at the periphery. The cell bodies of nociceptors cluster in the DRG, where each nociceptor extends two axons: one toward the periphery and the other to the dorsal horn of the spinal cord. At the periphery, nociceptor terminals transduce mechanical or chemical stimuli, including proinflammatory cytokines, inflammatory mediators such as those released from dysfunctional organelles, and mitochondrial ROS (mtROS), into pain signals via Ca²⁺/Na^+^ influx through TRP and Na_v_ channels. Under inflammatory conditions, NGF secreted by Møs, MCs, and FLSs synergizes with cytokines and mtROS to increase TRP/Na_v_ sensitization and SP expression, amplifying nociceptive signaling. Pain signals are transmitted to second-order spinal neurons through synaptic neurotransmitter release and then relayed to the brain for perceptual processing. AP action potential, SP substance P, ER endoplasmic reticulum, mtROS mitochondrial ROS, Mø macrophage, MC mast cell, FLS fibroblast-like synoviocyte, TRP transient receptor potential channel, Na_v_ voltage-gated sodium channel

After receiving nociceptive input from nociceptors, the axons of spinothalamic and spinoreticular cells—referred to as the spinothalamic and spinoreticular tracts—transmit these nociceptive signals upward to the brain.^176,185^ Both tracts crossover at the anterior white commissure of the spinal cord. The spinothalamic tract then diverges into two subdivisions: the anterior spinothalamic tract, which projects to the ventral posterior lateral nucleus and is thought to mediate pain discrimination, and the lateral spinoreticular tract. In contrast, spinoreticular tracts terminate in the reticular formation of the medulla and pons, contributing to the affective and motivational components of pain (Fig. 5).^156^

### Organelle dysfunction and osteoarthritis pain

Organelle dysfunction within the OA microenvironment contributes substantially to pain sensitization. Mitochondrial dysfunction, triggered by ROS and proinflammatory cytokines, leads to excessive ROS production and BAX/BAK-mediated mitochondrial outer membrane permeabilization, resulting in the release of mtDNA and ATP. These DAMPs activate PRRs, thereby driving inflammatory responses. Similarly, lysosomal dysfunction caused by hydroxyapatite crystal deposition induces LMP and the leakage of cysteine cathepsins, which activate the NLRP3 inflammasome. ER stress due to the accumulation of misfolded proteins activates the UPR, which in turn triggers inflammatory cascades. Collectively, these organelle-derived signals sensitize TRP and Na_v_ channels, amplifying nociceptive signaling in OA.

In addition to promoting inflammation, organelle dysfunction exacerbates OA pain by sensitizing TRP family members—particularly TRPV1, the most extensively studied in this context. TRPV1 is expressed on chondrocytes, intracellular organelles (mitochondria and the ER), and peripheral nociceptors.^186^ Under OA conditions, TRPV1 hyperactivation induces pathological Ca²⁺ influx, causing mitochondrial Ca²⁺ overload in both chondrocytes and nociceptors. This reduces the ΔΨm, leading to depolarization and creating a self-amplifying cycle in which ROS production and inflammation further sensitize TRP channels, ultimately potentiating OA pain signaling.^186,187^ Additionally, mitochondrial Ca²⁺ mobilization promotes the release of neurotransmitters from presynaptic nociceptors in the DRG, further amplifying nociceptive signal transduction in OA pain.^186^ Furthermore, TRPV1 hyperactivation directly mediates calcium efflux from the ER through its intrinsic ion channel, with the liberated Ca²⁺ being shuttled into mitochondria, where excessive uptake induces mitochondrial dysfunction. Moreover, aberrant Ca²⁺ regulation simultaneously triggers ER stress in both chondrocytes and peripheral nociceptors, activating the UPR pathway to amplify OA pain.^188–190^

TRPA1, which is localized to lysosomes in nociceptors, facilitates lysosomal Ca²⁺ release upon activation, enhancing neurotransmitter exocytosis and thus intensifying pain signaling.^191–193^ Additionally, nociceptor mitochondria absorb ~40% of the Ca²⁺ influx mediated by TRPM3 activation, potentially contributing to mitochondrial dysfunction and OA pain.^194^ However, the mechanistic links between TRPM3/TRPM8 activation and organelle dysregulation remain poorly understood and require further validation.

In the pain pathway of OA, excessive enhancement of OA pain signals leads to overactivation of neurotransmitter receptors, which causes dysfunction of organelles in second-order neurons. For example, disruption of Ca²⁺ homeostasis in nociceptors can result in increased release of neurotransmitters such as glutamate.^186,191–193^ The excessive release of glutamate causes overactivation of NMDA and AMPA receptors, leading to exaggerated Ca²⁺ influx that further results in mitochondrial depolarization and ER stress, potentially causing the death of second-order neurons.^195–197^ This may partially explain the association between OA and neurodegenerative diseases, as epidemiological evidence shows that individuals with OA have a 25% greater likelihood of being diagnosed with Parkinson’s disease (PD) or Alzheimer’s disease (AD).^197^

## Potential drugs and strategies to treat osteoarthritis

### Pharmacological treatment of osteoarthritis

Building on identified therapeutic targets for OA, pharmacological interventions can be structured around three pillars: anti-inflammation, analgesia, and the restoration of organelle homeostasis. Anti-inflammatory strategies aim to neutralize key cytokines (e.g., IL-1β, IL-6, and TNF-α) that drive cartilage degradation and synovial inflammation. Analgesic approaches target nociceptive signaling pathways to alleviate pain sensitization. Concurrently, therapies targeting mitochondrial dysfunction, ER stress, and lysosomal permeabilization in chondrocytes may disrupt the inflammation–organelle failure cycle. This tripartite framework offers a synergistic strategy to not only alleviate symptoms but also alter the OA disease trajectory.

#### Analgesics

Joint pain is the hallmark symptom of OA and a major contributor to reduced quality of life.^198^ The current management landscape includes physiotherapy, pharmacological treatment, and surgical interventions.^158,199^ Physiotherapy techniques such as heat or cold therapy enhance or restrict circulation to alleviate stiffness or reduce swelling. Lifestyle adjustments—such as weight loss and reduced joint loading—aim to decrease mechanical stress and associated pain.

Pharmacological agents commonly employed include NSAIDs, opioids, hyaluronic acid injections, corticosteroid injections, and growth factor injections such as platelet-rich plasma (PRP), which is derived from a patient’s blood and injected back into the affected joints to reduce pain. Despite their widespread use, these treatments fail to address the underlying causes of pain, and each treatment has limitations or adverse effects. For example, NSAIDs provide partial relief by inhibiting cyclooxygenase-1 (COX-1) and COX-2, which generate PGE_2_ but are ineffective in severe pain management and can lead to renal damage and gastrointestinal issues.^200^ Opioids offer modest benefits in chronic pain relief, but their use is limited by risks such as respiratory depression and addiction.^201^

Hyaluronic acid and corticosteroid injections require frequent administration because of their rapid in vivo metabolism,^35,202^ whereas the application of growth factors remains nonstandardized and requires further research to validate their efficacy.^35^ In end-stage OA, where pain becomes refractory to pharmacological treatments, surgical options, including total joint replacement, are often considered. While joint replacement has shown positive results in clinical settings, challenges such as joint infections, prosthesis dislocation, aseptic loosening, and the need for frequent revisions remain significant obstacles.^203^ Therefore, novel analgesic drugs with superior efficacy and fewer side effects are urgently needed to address OA-related pain.

Drawing upon the mechanisms underlying the etiology of OA pain delineated in the preceding section, the strategic targeting of TRP and Na_v_ channels, which play pivotal roles in the peripheral detection and transmission of noxious stimuli, alongside NGF, which regulates their expression, and the neurotransmitters implicated in the transmission of pain signals to second-order neurons, has emerged as a promising therapeutic strategy. These approaches hold significant potential for the development of innovative analgesics tailored to alleviate pain associated with OA.^204^

##### TRP family-based analgesics

TRP channels are key players in pain perception, as they transduce noxious stimuli into pain signals at the joint. These channels, along with the regulation of NGF, represent promising targets for therapeutic intervention in OA pain.

##### TRPV1-based analgesics

The TRPV1 channel is the most extensively studied TRP channel. Several small molecules that act as TRPV1 antagonists have been identified and are expected to form the basis for a new class of pain-relieving drugs. Notable compounds include AMG517, MK2295, and AZD1386.^205,206^ However, systemic administration of these antagonists in preclinical studies has been associated with adverse effects, such as hyperthermia or burn-like injuries, due to the loss of the sensation of noxious heat.^206,207^ The mechanism underlying these side effects is believed to involve proton activation of TRPV1 (at least in rat models),^206^ where antagonists blocking proton activation lead to hyperthermia, whereas compounds enhancing proton sensitivity can cause hypothermia.^208^ In response, efforts have been made to develop TRPV1 antagonists that retain analgesic effects while avoiding interference with proton activation. The second generation of these antagonists has shown promising results in clinical trials. For example, SB-705489 (GlaxoSmithKline) reduced dental pain in rats and has completed phase 2 trials for dental pain, followed by a phase 2 rectal pain trial with no hyperthermia reported.^205,206^ NEO6860 has demonstrated an analgesic effect without altering body temperature in knee OA patients,^162,209^ and XEN-D0501, although causing a slight increase in body temperature in healthy individuals, has been approved by the FDA for treating erythromelalgia.^208,210^ These findings suggest that TRPV1 antagonists could offer new insights into pain management for OA. However, caution is needed, as some TRPV1 antagonists may exhibit varying effects across species (e.g., JYL1421 causes hypothermia in rats but hyperthermia in dogs and cynomolgus monkeys),^206,208^ underscoring the need for further research to clarify their therapeutic potential in OA pain relief.

Interestingly, preclinical studies have shown that TRPV1 agonists may also exert analgesic effects, a phenomenon that contrasts with the actions of TRPV1 antagonists. Topical or site-specific capsaicin injections, which are potent TRPV1 agonists derived from chili peppers, alleviate OA pain.^205,211^ This paradox can be explained by prolonged or repeated exposure to capsaicin causing a large influx of calcium ions into nociceptors, activating calcineurin, a protein phosphatase that dephosphorylates TRPV1. This results in the desensitization of TRPV1-expressing nociceptors.^212,213^ However, the clinical application of capsaicin is limited due to its initial pain-inducing effects at the administration site, rapid metabolism (with a half-life of 1.6 h), and side effects such as hypothermia, pruritus, erythema, and papules.^205,213^

To overcome these limitations, efforts have focused on developing long-lasting, tolerable TRPV1 agonists that avoid the adverse effects of capsaicin. Resiniferatoxin (RTX), an ultrapotent capsaicin analog, can reversibly desensitize nociceptors for several months with a single low-dose injection and produces similar effects upon second administration. RTX may also provide permanent analgesia through irreversible ablation of nociceptors and is currently undergoing preclinical testing.^205,214,215^ CNTX-4975, a synthetic form of trans-capsaicin, considerably reduced joint pain in OA patients after a single 1.0 mg intra-articular injection, with effects lasting up to 24 weeks. In a phase 2 multicenter, double-blind trial, treatment-emergent adverse events were comparable to those of the placebo.^212^ Zucapsaicin, the cis-isomer of capsaicin, was approved in 2010 as a topical analgesic for severe knee pain in OA patients and is associated with fewer side effects than traditional capsaicin.^216^ Other TRPV1 agonists, including NE19550, MRD-652, and CPIPC, have shown promising analgesic effects in animal models,^205,213,216^ but their clinical efficacy in human OA remains to be fully established.

##### TRPA1-based analgesics

The TRPA1 channel is commonly characterized as a nonselective cation channel that detects noxious cold, mechanical stimuli, and irritant chemicals.^217,218^ While TRPA1 activation by noxious cold in healthy tissues is debatable, its role in cold hypersensitivity following tissue injury is well supported by multiple studies.^161^ In various OA rat models induced by complete Freund adjuvant (CFA), carrageenan, MIA, and monosodium urate, direct activation of TRPA1 by ROS, IL-1β, and TNF-α induces both mechanical and cold hypersensitivity. Notably, genetic deletion or pharmacological blockade of TRPA1 channels reverses allodynia without inducing the thermosensory or thermoregulatory disturbances typically observed with TRPV1 antagonism.^161,218^ These findings highlight the potential of TRPA1 antagonists as novel analgesics for OA pain management. Several promising TRPA1 antagonists, including HC-030031, AP-18, and A-967079, have been shown to reduce mechanical and cold hypersensitivity in animal models of CFA-induced inflammatory pain.^217,219^ However, a recent study reported conflicting results, in which systemic administration of HC-030031 did not alleviate ongoing pain in MIA-induced OA rats.^220^ This discrepancy may arise from the significant difference in TRPA1 expression between species, as TRPA1 is expressed at a much higher percentage (~80%) in human DRG neurons than in mouse DRG neurons, suggesting that the analgesic potency of TRPA1 antagonists may be underestimated in preclinical studies.^217,219^ Further clinical research is needed to elucidate the therapeutic potential of TRPA1 antagonists in OA pain management.

However, some TRPA1 agonists function similarly to TRPV1 agonists, desensitizing TRPA1-expressing neurons to produce analgesia. For example, systemic administration of atractylodin (ATR), a TRPA1 agonist, leads to sustained activation of TRPA1 (>1 h), which mitigates nocifensive behaviors induced by another TRPA1 agonist, allyl isothiocyanate (AITC).^221^ Additionally, intravenous injection of Ms 9a-1 markedly enhances TRPA1 responsiveness to agonists, thereby exerting an antinociceptive effect on CFA-induced inflammatory pain in rats.^222^ The mechanisms underlying the pro- or antinociceptive effects of certain TRPA1 agonists remain unclear. One hypothesis is that while pronociceptive TRPA1 agonists reversibly modulate TRPA1, antinociceptive agonists may irreversibly bind to cysteine residues near the cation-passing gate of TRPA1, thereby preventing channel reopening.^219^ Despite the analgesic potency of TRPA1 agonists, many challenges remain unresolved. For example, the administration of TRPA1 agonists may cause initial pain that is intolerable to patients, and TRPA1 agonists often suffer from issues such as poor chemical properties, low selectivity, or weak efficacy. Additional studies may provide further prospects for the use of TRPA1 agonists in OA pain treatment.

##### TRPM8-based analgesics

TRPM8 is recognized as a sensor for both innocuous cool (15–25 °C) and noxious cold (<15 °C) temperatures and is activated by compounds that elicit cool sensations, such as menthol, icilin, and eucalyptol.^223,224^ In contrast to the well-defined mechanisms of TRPV1 and TRPA1 channels in nociception, TRPM8 appears to play a bidirectional role in the pain pathway but exhibits a distinct anti- or pronociceptive pattern in specific pain conditions.^223^ For example, in the CFA model of inflammatory pain, mechanical and heat pain are substantially reduced following menthol injection, whereas pretreatment with TRPM8 antagonists or genetic deletion of TRPM8 abolishes this analgesic effect.^223,225^ Inflammatory conditions, including low pH and “inflammatory soup,” inhibit TRPM8 activity, and the mRNA expression of TRPM8 is reduced in human OA chondrocytes.^226^ These findings suggest that activation of TRPM8 may attenuate mechanical and heat allodynia in patients with inflammatory pain.^223^ Additionally, given that most nociceptors express TRPV1, the lack of coexpression of TRPM8 with TRPV1 suggests that TRPM8 does not interfere with the perception of mechanical and heat stimuli but blocks the transmission of evoked action potentials to second-order neurons.^223,227^

Conversely, TRPM8 activation can lead to cold-induced pain following injury and exacerbate cold hyperalgesia in patients with neuropathies.^223^ The application of high concentrations (30–40% wt/vol) of menthol has been shown to cause cold pain, cold allodynia, and cold hyperalgesia in human trials.^228,229^ These data suggest that patients with mechanical or heat pain as their primary symptom may be better treated with TRPM8 agonists at a low concentration, whereas individuals with cold-hyperalgesia may respond better to TRPM8 antagonists. Therefore, TRPM8 agonists are likely to be preferable therapeutic options for OA pain.

However, the use of TRPM8 agonists in OA pain treatment has rarely been reported. In a study involving 20 OA patients, the topical application of 3.5% menthol gel to OA knee joints resulted in no significant improvements in pain behavior compared with placebo.^230^ Additionally, coculture of menthol with OA chondrocytes increased the expression of MMPs and proinflammatory mediators in vitro.^226^ These findings suggest that further studies are needed to confirm the potential analgesic effects of TRPM8 agonists in treating OA pain.

##### TRPM3-based analgesics

TRPM3 is also a nonselective cation-permeable channel that is predominantly expressed on nociceptors, where it mediates nocifensive responses to the endogenous neurosteroid pregnenolone sulfate (PS) and noxious heat.^207,231,232^ Intraplantar injection of PS in mice elicits pain behaviors that are absent in TRPM3-deficient mice, suggesting that TRPM3 activation is critical for initiating pain sensation.^207,232^ Several studies have demonstrated that TRPM3 expression in DRG neurons increases under inflammatory conditions and that administration of TRPM3 antagonists in rodents abolishes TRPM3-mediated pain without affecting core temperature. Additionally, these antagonists reduce the responsiveness of TRPV1 and TRPA1 channels to their respective agonists, thereby enhancing the analgesic effects of TRPM3 blockade.^232,233^ Although TRPM3 antagonism may attenuate heat sensation, the perception of noxious heat remains intact as long as the heat sensor, TRPV1, remains functional,^218^ underscoring the potential of TRPM3 antagonists as promising analgesics for OA pain treatment.

However, research on TRPM3 antagonists as analgesics is still in its infancy. Plant-derived TRPM3 antagonists, such as isosakuranetin, ononetin, and primidone, suffer from suboptimal pharmacokinetic properties and lack target selectivity, hindering their therapeutic applicability in humans.^207^ Further research is needed to address these challenges, potentially by encapsulating these antagonists in appropriate delivery systems. Unlike other TRP channels, which are typically tetramers with a central cation-permeable pore,^234^ recent findings suggest that TRPM3 comprises an alternative pore. This alternative pore, synergistically activated with the central pore, intensifies pain sensation.^218,231^ This novel mechanism has sparked interest in targeting the alternative pore of TRPM3, and the concurrent blockade of both pores may enhance analgesia, offering a new avenue for OA pain management.

##### Voltage-gated sodium channel-based analgesics

Blockade of Na_v_ channels represents another promising strategy for alleviating OA pain, as these channels contribute to pain hypersensitivity in OA patients by initiating action potential firing and transmitting pain signals through Na^+^ influx in nociceptors.^235^ Current Na_v_ blockers used for pain management, such as local anesthetics (e.g., lidocaine), class I antiarrhythmics (e.g., mexiletine), antiepileptics (e.g., carbamazepine), and antidepressants (e.g., TCAs), have demonstrated significant efficacy in pain relief but also cause severe adverse effects, such as dizziness, sedation, convulsions, and cardiotoxicity, as these drugs nonselectively act on multiple Na_v_ subtypes related to motor and neurological functions,^236,237^ highlighting the need for selective Na_v_ blockers to minimize off-target effects.

Na_v_ subtypes, including Na_v_1.1 through Na_v_1.9, are expressed in various tissues and play pivotal roles in multiple physiological functions, necessitating careful consideration of potential side effects before specific subtypes are targeted.^237^ Blockade of Na_v_1.1, Na_v_1.2, and Na_v_1.3 channels, which are expressed primarily in the central nervous system (CNS), poses a risk of altered mental status or seizures.^236^ In contrast, the inhibition of Na_v_1.4 and Na_v_1.5 has been associated with muscular and cardiac channelopathies, respectively.^238^ Na_v_1.6 is expressed mainly in motor neurons, and blockade of this isoform leads to paresthesia and weakness.^239^ Na_v_1.9 is expressed in nociceptors and myenteric neurons in the gastrointestinal tract. Research on Na_v_1.9-specific blockers has been limited due to the rarity of clinical cases involving Na_v_1.9 mutations as well as challenges in developing Na_v_1.9-expressing cell models, which could explain the lack of promising Na_v_1.9-specific blockers despite clear evidence of Na_v_1.9 participation in pain signaling.^236,240^

The potential analgesic effects of Na_v_ channel blockers are often outweighed by their severe side effects. In contrast, specific inhibitors of Na_v_1.7 and Na_v_1.8 have shown the most promising results in pain treatment, with manageable adverse effects.^236^ Blockade of Na_v_1.7 results in manageable anosmia, whereas inhibition of Na_v_1.8 is associated with a risk of visual disturbances.^241,242^ Studies in OA mouse models have revealed upregulated expression of Nav1.7 and Nav1.8 in nociceptors innervating the knee joint, which is correlated with the pain hypersensitivity observed in these models.^243,244^ Several Nav1.7- and Nav1.8-specific blockers have been shown to attenuate secondary allodynia and improve motor function in murine OA models. For example, PF-04856264, a selective Na_v_1.7 blocker, substantially alleviates OA pain and upregulates type II collagen levels following intra-articular injection in a murine meniscectomy model, with similar results observed in MIA-induced OA rat models through oral administration.^245^ ProTx II, a tarantula toxin that selectively blocks Na_v_1.7, demonstrated no analgesic efficacy following intravenous or intrathecal injection but was found to upregulate the expression of type II collagen and aggrecan while reducing MMP13 and ADAMTS5 levels.^245–247^ A recently designed peptide, PTx2-3127, which is based on ProTx II, has shown efficacy in chronic and thermal pain in rat models via intrathecal administration, positioning it as a prospective novel analgesic.^248^

However, intra-articular injection of A-803467, a potent and selective Na_v_1.8 blocker, considerably reduced hindlimb disability and secondary allodynia in MIA-induced OA model rats.^247,249^ While specific Na_v_ blockers have shown promising analgesic effects in animal models of skeletal degenerative diseases, the pharmacological and toxicological profiles of these blockers in humans remain largely unknown, necessitating further studies before their clinical application.

##### NGF-based analgesics

NGF promotes the expression of the TRPV1 channel, Na_v_1.8 channel, CGRP, and SP, thereby enhancing pain sensation in OA patients. The inhibition of NGF signaling has emerged as a promising strategy for pain relief, with several clinical trials demonstrating that NGF antagonists outperform NSAIDs in terms of efficacy while avoiding severe side effects. These findings suggest that NGF is a prime target for the development of novel analgesics for OA pain management.^168,169,173,250^

Several strategies have been employed to inhibit NGF signaling, including (i) agents that capture free NGF to prevent its interaction with its receptor TrkA, (ii) agents that directly occupy the NGF-binding site, and (iii) agents that block TrkA receptors.^168,172^ NGF-capturing agents, such as humanized monoclonal antibodies, have shown remarkable specificity and potency in neutralizing NGF activity by directly binding to NGF.^168,169,172^ Notable examples include tanezumab (RN624), fasinumab (REGN475), and fulranumab, all of which have proven effective in alleviating OA pain. For example, tanezumab demonstrated superior analgesic effects to NSAIDs in treating hip or knee OA, although it was associated with a higher incidence of adverse events in a phase III trial conducted by Pfizer and Eli Lilly in 2019.^251^ Similarly, fasinumab and fulranumab considerably reduce pain and improve physical function in OA patients, with adverse events comparable to those of placebo in two phase II, randomized, double-blind, placebo-controlled trials.^252,253^

Additionally, several molecules that occupy NGF-binding sites and disrupt the interaction between NGF and its receptors, including ALE0540, PD90780, Ro 08-2750, Y1036, and PQC 083, have been investigated.^172,173^ Notably, systemic or spinal intrathecal administration of ALE0540 has demonstrated antiallodynic efficacy in animal models of neuropathic pain and thermally induced inflammatory pain.^254^ However, the analgesic effects of other molecules in this class remain to be fully elucidated.

The analgesic mechanisms of both NGF-capture agents and NGF-binding site antagonists rely on blocking NGF activity. However, this blockade may inadvertently disrupt homeostatic cellular responses mediated by NGF-p75^NTR^ interactions, potentially complicating long-term use.^173^ Consequently, selective inhibitors of TrkA, which is a high-affinity NGF receptor, may offer greater clinical benefits. Nonetheless, TrkA is widely expressed in cholinergic neurons of the CNS, raising concerns about potential side effects in the CNS. To mitigate these risks, the use of TrkA inhibitors should ideally be restricted to peripheral applications.^255^

Most TrkA inhibitors target the ATP-binding site, a highly conserved motif shared among Trk family members, which may inadvertently inhibit other Trk receptors and exacerbate the CNS or other adverse effects.^168,255,256^ To address this, numerous small molecules with high selectivity for TrkA have been developed by targeting less conserved ligand-binding regions. An exemplary compound in this category is PF-06273340, a potent TrkA inhibitor with low cytotoxicity. PF-06273340 inhibits TrkA by 98% at a concentration of 1 μM and can be effluxed by P-glycoprotein (P-gp), a transporter that prevents its entry into the brain, thereby reducing the risk of side effects in the CNS.^255,256^ In preclinical models, PF-06273340 has shown efficacy in alleviating pain in an ultraviolet burn-induced hyperalgesia model of inflammatory pain, suggesting that it is a promising candidate for clinical application in OA pain management.^256^

Another promising TrkA inhibitor is AR786, which has good oral bioavailability and low CNS penetration. Chronic oral administration of AR786 effectively prevented pain in MIA-induced OA model rats.^257^ As research into TrkA-selective inhibitors continues to expand, these compounds hold considerable promise for addressing unmet medical needs in OA pain treatment.

##### Neurotransmitter-based analgesics

Neurotransmitters play a critical role in nociceptive transmission. Targeting their transport or receptor activity can reduce nociceptive signaling and offer analgesic benefits. SP is one such neurotransmitter involved in pain transmission, and its receptor, NK1R, has emerged as a potential therapeutic target. Despite the success of various NK1R antagonists in animal models, human trials have not demonstrated significant analgesic effects.^183,258^ This discrepancy may be due to species differences or the limited impact of blocking a single SP signaling pathway amid the broader network of neurotransmitters involved in nociception.^258^

In addition to SP, CGRP is another important neurotransmitter in nociception. This 37-amino acid peptide binds to an atypical receptor composed of CLR, RAMP1, and RCP, which is located on both nociceptors and second-order neurons.^181^ CGRP sensitizes nociceptors and transmits pain signals via its receptor, suggesting that blocking CGRP receptors could offer analgesic potential. While CGRP receptor antagonists are well established in migraine treatment, their role in OA pain remains less explored. However, two studies have shown that CGRP receptor antagonists considerably reverse nociceptor sensitization and pain-related behaviors in OA animal models. For example, the subcutaneous injection of BIBN4096BS in MIA-induced OA rats provided morphine-like analgesia within 4 h.^259^ Intra-arterial injection of CGRP 8-37 reversed joint nociceptor mechanosensitization in both MIA-induced and meniscectomy OA models.^260^ These studies confirmed that CGRP receptor antagonists block receptors on joint nociceptors to prevent sensitization. However, whether they also inhibit nociceptive transmission by acting on second-order neurons remains to be clarified.

Glutamate is another central neurotransmitter involved in nociceptive transmission. Pharmacological blockade of glutamate receptors, including iGluRs and mGluRs, has demonstrated analgesic effects in both preclinical and clinical studies.^261,262^ The iGluR family includes N-methyl-D-aspartate (NMDA) receptors, α-amino-3-hydroxy-5-methylisoxazole-4-propionic acid (AMPA) receptors, kainate (KA) receptors, and δ (GluD) receptors. GluD receptors differ in that they require the intracellular ligand D-serine for activation rather than glutamate, and their role in nociception is still under investigation,^263^ so they are not covered here.

Excluding GluD, all iGluRs are ligand-gated ion channels permeable to Ca²⁺, Na^+^, and K^+^, causing depolarization of second-order neurons upon activation.^264^ NMDARs are heteromeric complexes typically containing the NR1 subunit and one or more NR2A-D subunits.^265^ While most subunits are ubiquitously expressed in the CNS, NR2B-containing NMDA receptors are more selectively distributed in the forebrain and superficial dorsal horn of the spinal cord.^265,266^ Traditional nonselective NMDA antagonists—such as ketamine, memantine, and MK-801—act on all NMDA subunits,^267^ but they can cause adverse effects such as neurotoxicity, motor impairment, learning impairment, and psychotomimetic symptoms at or near the antinociceptive dosage.^268,269^ To avoid these side effects, selective antagonists of NR2B-containing NMDA receptors represent a novel therapeutic option that might be devoid of the most prominent side effects of nonselective NMDA antagonists while retaining analgesic efficacy.

Ifenprodil is a potent and selective NR2B-containing NMDA receptor antagonist. Spinal administration of ifenprodil alleviates mechanical allodynia and neuropathic pain without cognitive or psychotomimetic side effects.^265,266^ Other newly developed NR2B-containing NMDA receptor antagonists, including CP101606, Ro256981, and CI1041, have shown antinociceptive effects without CNS side effects.^266^ However, the pharmacological properties of these drugs have never been tested in animal models of OA pain; more preclinical data will be necessary to assess their feasibility in relieving OA pain.

AMPA receptors, another iGluR subtype, are composed of GluR-A-D (also known as GluR1-4).^270^ GluR-B has an arginine residue at the pore-forming segment, which introduces additional positive charges into the pore that inhibit Ca²⁺ influx,^271^ increasing its antinociceptive properties and limiting its potential as a drug target. Unlike GluR-C and GluR-D, Ca²⁺-permeable AMPA receptors are predominantly composed of homomeric GluR-A subunits at second-order neurons of the spinal dorsal horn,^270^ and several animal studies have demonstrated that the activation of GluR-A-containing AMPA receptors leads to long-lasting nociceptive hypersensitivity,^270,272^ making GluR-A the most attractive target among other subunits in analgesia. However, selective antagonists for GluR-A remain elusive. Nonselective AMPA receptor antagonists, such as NBQX, have yielded mixed results in OA pain models. For example, in antigen-induced arthritis (AIA) rats, intra-articular injection of NBQX, an AMPA/KA receptor antagonist, reduced pain-related behaviors within 2 days,^273^ but it had no analgesic effect on posttraumatic OA rats.^274^ More data are needed to demonstrate the effectiveness of AMPA receptor antagonists in analgesia and the role of GluR-A antagonists in OA pain treatment.

Kainate receptors, the final members of the iGluR family, consist of five subunits, including GluK1-5 (formerly known as Glu5-7, KA1, and KA2).^275^ In contrast to the restricted localization of the NMDA receptor and AMPA receptor on postsynaptic neurons, the kainate receptor is distributed on both presynaptic and postsynaptic neurons at the spinal dorsal horn.^275,276^ Kainate receptors on presynaptic neurons (including nociceptors) mediate the release of glutamate, whereas those on postsynaptic neurons (including second-order neurons) contribute to nociceptive transmission by binding to glutamate.^277^ Among the kainate receptor subunits, GluK1 is predominantly expressed in nociceptors within the DRG, and it appears to play a crucial role in nociceptive transmission, as the kainate receptor exhibits an absolute requirement for GluK1, for which kainate receptor-mediated currents are not detected in GluK1 knockout DRG neurons.^277,278^ Recent studies have extensively explored the efficacy of several GluK1 antagonists in analgesia, and some have shown promising results in preclinical trials. MSVIII-19 (8,9-dideoxy-neodysiherbaine) is a potent GluK1 antagonist. An elegant experiment conducted by Chang-Shen Qiu and colleagues revealed that intrathecally administered MSVIII-19 reduced thermal hypersensitivity in animal models of inflammatory pain and both thermal hyperalgesia and mechanical hypersensitivity in a chronic construction injury model of neuropathic pain.^275^ LY467711 and LY525327 are two orally effective prodrugs that are metabolized into two selective GluK1 antagonists called LY458545 and LY457691 in vivo. Compared with morphine and ibuprofen, the oral administration of these two prodrugs has shown preferable analgesic efficacy in three classical pain models, including formalin-induced late-phase paw-licking pain, carrageenan-induced thermal hyperalgesia, and capsaicin-induced mechanical hyperalgesia.^279^

Finally, mGluRs, another critical family of glutamate receptors, also play crucial roles in the pain pathway. mGluRs can be divided into three groups on the basis of amino acid homology: group I (mGluR1 and mGluR5), group II (mGluR2 and mGluR3), and group III (mGluR4, mGluR6, mGluR7, and mGluR8).^280^ Like iGluRs, mGluRs are involved in transmitting pain signals, but the functions of different groups vary on the basis of their distributions. Group I mGluRs are expressed in both pre- and postsynaptic neurons and initiate action potential firing in second-order neurons when activated, thus contributing to pain signal transmission. In contrast, group II and III mGluRs are located predominantly at presynaptic terminals of nociceptors, where they act as autoreceptors to suppress nociceptor excitability upon activation.^281,282^ Given their distinct roles, targeting specific subtypes of mGluRs, such as blocking group I mGluRs or activating group II/III mGluRs, offers promising therapeutic strategies for OA pain relief. Numerous studies have demonstrated the analgesic potential of group I mGluR antagonists and group II/III mGluR agonists. The spinal administration of group I mGluRs such as LY367385 and AIDA has been shown to reverse thermal hyperalgesia in rats with knee inflammation without affecting basal perception.^283^ Activating group II mGluRs with the agonist APDC blocks nociceptor hyperexcitability without affecting basal membrane excitability.^281^ Other group II mGluR agonists (LY379268, LY354740, LY389795, and LCCG1) have demonstrated analgesic properties in many inflammatory pain models (capsaicin-treated and formalin-treated models),^280^ whereas group III mGluR agonists, such as ACPT-I and PHCCC, inhibit nociceptive behaviors in rats subjected to formalin tests and mechanical hyperalgesia in various models of inflammatory pain (carrageenan-treated and monoarthritic rats).^282^ These findings underscore the importance of mGluRs in nociceptive transmission and highlight their potential as therapeutic targets for OA pain treatment.

#### Anti-inflammatory effects

The devastating inflammation observed in the OA joint is closely linked to the massive release of proinflammatory cytokines, which contribute to hallmark clinical manifestations, such as swelling, synovitis, and inflammatory pain. Catabolic events elicited by these inflammatory cytokines further aggravate the inflammatory milieu, thereby exacerbating OA progression. Targeting key cytokines involved in OA progression—specifically IL-1, IL-6, and TNF-α—through pharmacological blockade may provide a foundation for the development of novel therapeutic strategies. This section systematically reviews potential therapeutic targets identified within the signaling pathways of IL-1, IL-6, and TNF-α, discusses corresponding therapeutic agents, and highlights the challenges involved in managing OA-related inflammation and preventing disease progression.

##### IL-1-targeting pharmacological agents

IL-1 exerts a variety of negative effects on the pathogenesis of OA, inducing both local and systemic inflammation. It upregulates MMPs and ADAMTS, inhibits the synthesis of the proteoglycans aggrecan and type II collagen, and disrupts the balance between osteoblasts and osteoclasts. These effects collectively lead to tissue degradation, bone erosion, and osteophyte formation. Given its centrality in OA pathology, IL-1 signaling represents a promising therapeutic target for mitigating disease progression. A deeper understanding of IL-1 signaling mechanisms is essential for identifying potential therapeutic interventions. On the basis of the involvement of IL-1 signaling in OA progression, therapeutic strategies targeting IL-1 can be categorized into three main approaches: (i) agents that antagonize IL-1RI, (ii) agents that neutralize circulating IL-1, and (iii) agents that inhibit the maturation of IL-1.

##### IL-1RI antagonists

IL-1Ra exerts anti-inflammatory effects by competitively antagonizing IL-1 at IL-1RI, thereby blocking downstream proinflammatory signaling without receptor activation. This mechanism suggests therapeutic potential for OA, a hypothesis supported by epidemiological evidence demonstrating an inverse correlation between serum IL-1Ra concentrations and the radiographic severity of knee OA.^284,285^ Despite its anti-inflammatory properties, the therapeutic application of IL-1Ra in OA remains limited by its short intra-articular half-life and suboptimal therapeutic efficacy. Current research focuses on bioengineering strategies to increase IL-1Ra pharmacokinetics. For example, Khaled et al. engineered a PLGA microsphere encapsulating IL-1Ra, demonstrating that intra-articular administration preserved the inherent cartilage-protective bioactivity of IL-1Ra while substantially enhancing disease-modifying efficacy in a rat model of ACLT-induced OA.^286^ Similarly, Shikhar et al. designed an IL-1Ra through cationic carrier-mediated modification, enabling electrostatic targeting to the negatively charged aggrecan-glycosaminoglycan matrix within cartilage. While unmodified IL-1Ra failed to attenuate IL-1-induced cartilage degradation over 16 days, the modified IL-1Ra considerably suppressed induced glycosaminoglycan loss and nitrite release concomitant with improvements in chondrocyte metabolism and viability.^287^

Despite promising preclinical efficacy, IL-1Ra protein-based therapies remain limited by the need for repeated administration. Gene therapy has emerged as a strategic alternative to circumvent this challenge, offering sustained IL-1Ra expression and novel therapeutic insights for OA management.^288^ Deng et al. developed a polyplex nanomicelle-based delivery system for mRNAs encoding IL-1Ra. Compared with the control, a single intra-articular injection of this construct in a rat model of temporomandibular joint OA alleviated pain and suppressed OA progression for 28 days, as evidenced by reduced pain-related behaviors and attenuated cartilage degradation.^289^ Alan et al. employed helper-dependent adenovirus (HDAd)-mediated intra-articular gene therapy to deliver the IL-1Ra-encoding gene, effectively preventing pathological progression in murine and equine OA models.^290^ Frisbie et al. designed an adenoviral vector expressing equine IL-1Ra DNA (Ad-EqIL-1Ra), which was administered via intra-articular injection in equine OA models. This intervention reduced joint pain-related lameness and pathological changes while exerting protective effects against proteoglycan loss. Notably, therapeutic efficacy was observed even when treatment was initiated 14 days post-OA induction, indicating potential utility in addressing established joint trauma.^288^

While these preclinical advancements in IL-1Ra delivery optimization underscore its therapeutic promise, clinical translation necessitates confronting inherent pharmacokinetic challenges. The translational gap between preclinical efficacy and clinical applicability becomes evident in human trials, where even mechanistically validated strategies face hurdles such as short half-lives and suboptimal biodistribution. For example, anakinra, a recombinant IL-1Ra, has been approved by the FDA for treating rheumatoid arthritis (RA).^56,64,291^ Clinical trials have demonstrated its safety and tolerability, with only rare cases of anaphylaxis. However, owing to its relatively short half-life of 4–6 h, daily subcutaneous injections are required to maintain therapeutic efficacy, which can lead to frequent injection-site reactions.^49,50,64^ AMG108, a humanized monoclonal antibody that targets IL-1RI, is still in clinical development for OA and RA treatment.^56^ Phase II trials have shown a favorable safety profile, with intravenous or subcutaneous administration, but the clinical efficacy has been limited, with only modest improvements observed in RA patients and no significant effects in OA patients to date.^292,293^

##### IL-1 neutralizing agents

Canakinumab is a humanized monoclonal antibody that selectively neutralizes IL-1β, with no effect on IL-1α-driven inflammation.^56,291^ With a long average half-life of 26.1 days,^51^ canakinumab is administered every 4–8 weeks via subcutaneous injection. It is approved for treating cryopyrin-associated periodic syndrome (CAPS) and systemic-onset juvenile idiopathic arthritis.^49,56,64^ Rilonacept, a dimeric fusion protein comprising the Fc region of human IgG1 and the extracellular IL-1 binding site of IL-1RI and IL-1RAcP, can neutralize circulating IL-1α, IL-1β, and IL-1Ra.^51,56^ The safety profile of rilonacept is still inconclusive due to limited clinical data, but it appears comparable to that of anakinra.^50,56^ It requires weekly subcutaneous injections and has been approved by the FDA for treating CAPS, recurrent pericarditis, and IL-1Ra deficiency (DIRA). However, indiscriminate neutralization of IL-1Ra may hinder the therapeutic efficacy of rilonacept.^56,291^ To optimize clinical outcomes, the modulation of rilonacept to preferentially neutralize IL-1α and IL-1β while preserving the affinity of IL-1Ra may be beneficial. Recent studies by Yusha Wang et al. revealed a de novo variant, p.Lys131Glu (K131E), in IL-1R1 that reduces its affinity for IL-1Ra. They further designed a novel IL-1-neutralizing agent, rilabnacept, upon the modulation of rilonacept with the K131E mutation, which showed promise in preclinical models of IL-1-driven diseases.^291^

In addition to the agents already in clinical use, several other neutralizing IL-1 agents are currently under investigation. These include bermekimab, a naturally occurring monoclonal antibody that neutralizes IL-1α; gevokizumab, a humanized monoclonal antibody that targets IL-1β; and lutikizumab, a dual-targeting antibody that blocks both IL-1α and IL-1β.^56^ These emerging therapies offer promising alternatives or adjuncts to existing treatments, potentially enhancing the ability to modulate the IL-1-mediated inflammatory pathways in OA. As clinical development advances, the efficacy and safety profiles of these agents will be key in determining their future role in managing IL-1-mediated diseases, including OA.

##### Caspase-1 inhibitors

VX-740 (also known as pralnacasan) is an orally active, reversible caspase-1 inhibitor developed in cooperation with Vertex Pharmaceuticals and Aventis.^294^ In murine OA models, high-dose VX-740 (50 mg/kg) substantially alleviated inflammation and reduced cartilage degradation and bone erosion indicators by 59% and 84%, respectively.^295^ The subsequent phase I clinical trials demonstrated favorable tolerability of VX-740 with 50% oral bioavailability. In phase II clinical trials involving 285 RA patients, VX-740 showed a trend toward symptom improvement but failed to reach statistical significance. Phase III trials for RA and OA treatment were discontinued because of the liver toxicity observed in long-term animal studies.^50,294^ VX-765 (belnacasan), another reversible caspase-1 inhibitor developed by Vertex Pharmaceuticals, has greater potency than VX-740.^50^ Phase I clinical trials have shown that VX-765 meets pharmacokinetic and pharmacodynamic objectives, while phase II trials for psoriasis were completed in 2005, although no further updates have been published.^294^ A recent study demonstrated that oral administration of VX-765 considerably alleviated inflammation and joint tissue damage in a murine OA model,^296^ suggesting its potential as a novel anti-inflammatory agent for OA treatment.

To summarize, currently FDA-approved anti-IL-1 drugs—such as anakinra, canakinumab, and rilonacept—are associated with common biologic-related side effects, including injection-site reactions, reversible neutropenia, and an increased risk of bacterial infections.^49^ Despite encouraging preclinical and early clinical findings, monotherapy with IL-1-targeting agents (such as anakinra, canakinumab, rilonacept, AMG 108, and VX-740) has not yielded substantial clinical improvements in RA or OA patients, although some trends toward relief have been noted.^48^ These findings suggest that exclusive inhibition of IL-1β may be insufficient for effective OA treatment. Combination therapies targeting IL-1β along with other anti-inflammatory agents may enhance the therapeutic efficacy of reducing OA-associated inflammation.

##### TNF-α-targeting pharmacological agents

As discussed earlier regarding TNF-α-mediated signaling in OA pathogenesis, therapeutic strategies targeting TNF-α can be categorized into three main approaches: (i) neutralizing both soluble and transmembrane sTNF-α and tmTNF-α; (ii) antagonizing TNFR-1 and TNFR-2 receptors; and (iii) inhibiting TACE, which reduces sTNF-α production and may partially prevent TNFR-1-expressing (including chondrocytes) apoptosis induced by the binding of sTNF-α and TNFR-1. However, since tmTNF-α also binds TNFR-1 with high affinity and TACE also cleaves TNFRs into soluble forms that act as natural TNF-α antagonists,^297,298^ TACE inhibition may yield minimal or even counterproductive effects. For example, marimastat, a TACE inhibitor, suppressed LPS-induced sTNF-α in mice but only slightly delayed LPS-induced lethality.^298^

Traditional TNF-α-targeting agents approved by the FDA include infliximab, etanercept, and adalimumab.^68,298^ Infliximab is a chimeric monoclonal antibody composed of a murine TNF-α-binding site fused with the Fc region of human IgG1, enabling it to bind specifically to TNF-α and block its interaction with TNFRs.^68,299,300^ When infliximab is administered intravenously with a half-life of 8–9.5 days, it is approved for treating Crohn’s disease (CD), ankylosing spondylitis (AS), psoriatic arthritis (PsA), psoriasis (PSO), and RA.^68,301^ However, its murine component may elicit human anti-chimeric antibodies (HACAs), reducing therapeutic efficacy—an effect that can be ameliorated with the coadministration of methotrexate.^301^ Adalimumab is a fully human IgG1 monoclonal antibody with a similar mechanism but lower immunogenicity.^68,298^ Nevertheless, long-term use may still lead to the development of anti-adalimumab antibodies.^301^ When administered subcutaneously with a half-life of 10–13 days, it is approved for PsA, PSO, AS, CD, juvenile idiopathic arthritis (JIA), uveitis, and RA.^68^

Etanercept is a fusion protein that combines the extracellular region of TNFR-2 and the Fc region of IgG1. It is administered subcutaneously with a half-life of 3–3.5 days and is approved for PsA, PSO, AS, and RA.^68,299,301^ All these agents contain an IgG1 Fc region, enabling them to induce lysis of tmTNF-α-producing cells (including chondrocytes) via complement-dependent cytotoxicity and antibody-dependent cell-mediated cytotoxicity, potentially exacerbating OA progression.^302^

This issue is addressed by certolizumab pegol, a recently approved TNF-α-targeting agent. It is a humanized monoclonal antibody lacking the Fc region and consists of a human IgG1 Fab fragment with a murine anti-TNF-α amino acid sequence covalently linked to a polyethylene glycol moiety.^68,299^ This PEGylation enhances pharmacokinetics, yielding a subcutaneous half-life of ~14 days. In CD, PSO, PsA, AS, and RA, certolizumab pegol avoids Fc-mediated cytotoxicity.^68^ However, similar to other TNF-α antagonists, it may still induce immunogenicity and antibody formation, limiting its long-term use. These agents also increase the risk of infections (particularly tuberculosis), malignancies, systemic lupus erythematosus-like syndromes, and demyelinating diseases.^300^ The latter two are thought to be associated with disrupted TNF-α-TNFR-2 signaling (discussed below).^299^

Recent studies underscore the role of TNFR-2 in TNF-α-associated autoimmunization. Approximately 30% of regulatory T (T_reg_) cells—key mediators in the suppression of immune overactivation—express TNFR-2 but not TNFR-1.^303^ The binding of TNF-α and TNFR-2 initiates expansion, prolonged survival, and increased phenotypic stability of T_reg_ cells in humans, thus spurring research into the use of TNFR-2 agonists as potential therapies for autoimmune diseases.^304^ However, paradoxically, the proportion of T_reg_ cells increases in most autoimmune diseases, including RA, following TNF-α inhibition therapy. This phenomenon may be explained by certain anti-TNF-α agents, such as adalimumab, which conversely increase the expression of tmTNF-α on monocytes, thereby promoting the interaction between tmTNF-α and TNFR-2.^305^ The change in the proportion of T_reg_ cells caused by nonselective blockade of TNF-α-TNFR-2 signaling might partially account for the autoimmune-related adverse effects and infections observed with anti-TNF-α monoclonal antibody therapy.

However, the proinflammatory effects are predominantly mediated by TNFR-1, suggesting that selective antagonism of TNFR-1 may be a promising strategy for treating OA, as it preserves TNFR-2-mediated immunoregulation and potentially prevents the side effects typically associated with monoclonal antibody therapy.^306^ Several attempts have been made to inhibit TNFR-1, such as R1antTNF—a mutant form of human TNF-α that selectively blocks TNFR-1 by competing with TNF-α for the binding site—showing promise in OA inflammation treatment. However, its therapeutic effects are limited by its short half-life (~10 min) in murine plasma when it is administered intravenously. PEGylation of R1antTNF extended its half-life to 12 h without compromising bioactivity. Both prophylactic and therapeutic use of PEGylated R1antTNF showed comparable efficacy to etanercept in reducing the severity of arthritis in a murine CIA model without impairing the immune response to infections—an issue observed with etanercept.^307^

Atrosimab, a monoclonal antibody targeting TNFR-1, considerably reduces the development and severity of arthritis in murine models, showing effects comparable to those of infliximab, etanercept, and certolizumab pegol in both prophylactic and therapeutic settings.^308^ Other selective TNFR-1 antibodies (IZI-06.1, TNFR1-AlbudAb, Nb 70 and Nb 96), as well as small molecules (PMG, R1), have demonstrated the ability to prevent TNFR-1-induced apoptosis and cytokine production in certain cell types without compromising TNFR-2-mediated immune regulation, suggesting promising therapeutic strategies for OA.^306^ However, these agents still rely on a similar mechanism of blocking TNFR-1 by competing for the same TNF-α binding site, which may limit their therapeutic efficacy owing to the high affinity of both sTNF-α and tmTNF-α for TNFR-1.

Recent studies have also identified noncompetitive TNFR-1 inhibitors, such as DS41, DSA11, and Zafirlukast, which bind to distinct sites on TNFR-1 to stabilize its nonfunctional state, thereby blocking downstream signaling without interfering with TNFR-2 regulation.^306^ In conclusion, current pharmacological anti-TNF-α agents (infliximab, etanercept, adalimumab, and certolizumab pegol) have demonstrated substantial clinical efficacy in treating various forms of arthritis, particularly RA. With a deeper understanding of TNF-α signaling, selective TNFR-1 antagonists have been developed to minimize the side effects of traditional anti-TNF-α therapies while retaining their anti-inflammatory properties. These agents have shown promising therapeutic effects in preclinical arthritis models. However, their efficacy in OA treatment remains underexplored, and further research is needed to evaluate the potential of anti-TNF-α therapy in OA management.

##### IL-6-targeting pharmacological agents

The IL-6 pathway has emerged as a pivotal factor in the pathogenesis of various diseases, including OA. As such, targeting IL-6 represents a promising anti-inflammatory strategy for OA treatment. IL-6 has three key binding sites: site I, site II, and site III. Through site I, IL-6 binds to either mIL-6R or sIL-6R to form a membrane-bound or circulating complex, which subsequently interacts with dimerized gp130 via sites II and III, initiating classic or trans-signaling.^309^ As discussed in the previous section, this IL-6 pathway is a central focus of therapeutic intervention.

Therapeutic strategies targeting the IL-6 pathway can be summarized into several categories: (i) blockade of gp130, (ii) blockade of circulating IL-6, (iii) blockade of IL-6R, (iv) inhibition of ADAM10/ADAM17, (v) inhibition of sIL-6R transcription, and (vi) inhibition of intracellular participants such as JAKs and STAT3. While gp130 serves as a signaling receptor for multiple cytokines, its blockade could disrupt cytokine homeostasis. Animal studies have shown that gp130-deficient mice are nonviable,^309,310^ making gp130 inhibition an undesirable approach.

Conventional therapies primarily target circulating IL-6 or IL-6R via monoclonal antibodies. For example, tocilizumab is a humanized anti-IL-6R monoclonal antibody created by grafting the complementarity-determining regions of a mouse anti-human IL-6R antibody into human IgG1κ.^311^ It blocks both mIL-6R and sIL-6R by competing for site I on IL-6R. Tocilizumab has shown efficacy in protecting against joint destruction in RA treatment and is approved for treating RA, juvenile idiopathic arthritis, and other IL-6-related diseases.^310^

Other monoclonal antibodies targeting IL-6 (e.g., olokizumab, siltuximab, sirukumab, and clazakizumab) and IL-6R (e.g., sarilumab, satralizumab, and vobarilizumab) have also been shown to be effective in reducing joint inflammation and destruction.^310,311^ However, an increased risk of bacterial infections is commonly reported,^83,310^ likely due to the role of IL-6 in plasma-cell differentiation and antibody production.^310,311^ Further studies suggest that this risk is associated primarily with classic-signaling^312–314^, whereas trans-signaling drives catabolic activity in chondrocytes.^315^ For example, infections are the most common adverse events in tocilizumab-treated hand OA patients, whereas the trans-signaling-specific antagonist olamkicept has shown minimal immune impact and preserved bone repair in a murine femur osteotomy model.^316,317^ This distinction has fueled interest in trans-signaling inhibitors.

Olamkicept (also known as sgp130Fc), a humanized monoclonal antibody composed of two sgp130 proteins fused to the Fc region of human IgG, is designed to compete for sites II and III on IL-6 with membrane-bound gp130.^309,318^ While this configuration theoretically blocks both classic and trans-signaling by neutralizing the IL-6-sIL-6R and IL-6-mIL-6R complexes, animal studies have shown that olamkicept selectively inhibits trans-signaling unless it is administered at high doses,^309,310,313,314^ suggesting that sgp130 may bind to IL-6-sIL-6R with increased affinity. Olamkicept has demonstrated promise in preclinical arthritis models, with intra-articular injections considerably reducing joint inflammation and cartilage damage in RA mice, supporting the potential of selective IL-6 trans-signaling inhibition for OA.^319^

However, sgp130 is also involved in other cytokine pathways, such as the IL-11 pathway, raising concerns about disruption of cytokine homeostasis.^318^ A recent study addressed this issue by developing cs-130Fc, a monoclonal antibody that combines the binding region of sgp130 (domains D1–D3), a nanobody (VHH6) that targets the IL-6-sIL-6R complex, and a human IgG Fc region. Cs-130Fc was shown to double the potency of olamkicept in inhibiting IL-6 trans-signaling while reducing IL-11 trans-signal inhibition by eightfold,^313,318^ offering new insights into anti-inflammatory therapy for OA.

Targeting the mIL-6R-cleaving enzymes ADAM10 and ADAM17 or inhibiting the transcription of sIL-6R to prevent the formation of IL-6–sIL-6R complexes represents another alternative strategy for inhibiting trans-signaling. However, a recent study suggested that the downregulation of ADAM10 and ADAM17 promotes the differentiation of murine bone marrow cells and human blood cells into osteoclasts, which may exacerbate bone resorption in OA patients.^320^ In contrast, inhibiting sIL-6R transcription may be insufficient for blocking IL-6R trans-signaling, as only ~15% of sIL-6R production is derived from RNA transcription, and no available agents targeting this process have been reported.^313^

Although targeting IL-6 trans-signaling to inhibit inflammation while preserving host defense against bacterial infections is generally considered the most promising approach for anti-IL-6 therapy, a recent study revealed that IL-6R antibodies, the trans-signaling inhibitor sgp130, and the cleavage enzyme inhibitor TAPI2 did not reduce the expression of IL-6 or inflammatory mediators (NO, PGE_2_, and MMPs) in chondrocytes or increase chondrocyte viability in a late-stage human knee OA model. However, an intracellularly active gp130 inhibitor, SC144, substantially improved these conditions.^315^ These findings suggest that agents targeting extracellular IL-6 signaling may have limited therapeutic effectiveness once intracellular signaling is established in late-stage OA.^83,315^

Therefore, blocking intracellular signaling components, such as JAKs (especially JAK1, which predominantly activates IL-6) or STAT3, may be preferable options for advanced OA treatment.^321^ Several JAK and STAT3 inhibitors have been developed, including pan-JAK inhibitors (tofacitinib and baricitinib), JAK1-selective inhibitors (filgotinib), TYK2-selective inhibitors (deucravacitinib), and STAT3 inhibitors (OPB-31121, OPB-51602).^310^ However, the development of IL-6 intracellular inhibitors is associated with several challenges, including low cell membrane permeability,^309^ disruption of cytokine homeostasis due to the involvement of JAK and STATs in other cytokine pathways,^313^ and the nonselective blockade of both classic and trans-signaling (as both intracellular mechanisms are currently understood to be identical).^309,310,312^

Targeting global intracellular signaling could result in side effects associated with classic-signaling, such as increased susceptibility to bacterial infections. Recent studies have shown that the administration of STAT3 inhibitors is customarily accompanied by side effects of peripheral neuropathy, and only limited efficacy in improving cartilage lesions has been reported in OA mice.^76,310^ In conclusion, the selective inhibition of IL-6 trans-signaling remains the most promising therapeutic approach for the prevention or early treatment of OA until novel intracellular signaling discriminating targets or refined dosage forms of JAK and STAT3 inhibitors with reduced side effects are developed, which may provide new insights for advanced OA treatment (Table 1).

**Table 1:** Summary of potential OA analgesics

Genre	Targets	Action	Drug	Tested in OA	Preclinical or clinical effects	Adverse effects	Ref.
TRP family	TRPV1	Antagonists	AMG517	No	Demonstrated efficacy in rodent models of inflammatory pain	Transient drug-related body temperature increased in rodents, dogs, and monkeys	^419^
			MK2295	No	Phase 2 clinical trial for dental pain was completed (NCT00387140), but no results were released	Loss of noxious heat sensation in human trials	^420^
			AZD1386	Yes	Demonstrated no KOA pain relief in a phase IIb dose-finding trial	Loss of noxious heat sensation in human trials	^421^
			SB-705489	No	Reduced dental pain in rats Phase 2 dental pain clinical trial completed Phase 2 rectal pain trial completed with no hyperthermia reported	No hyperthermia or hypothermia adverse events in human trials were reported	^205,206^
			NEO6860	Yes	Demonstrated analgesic trend in a phase 2 trial for KOA patients	Feeling hot, headache, nausea, dizziness, fatigue, hypoaesthesia, and increased blood pressure were observed in human trials	^209^
			XEN-D0501	No	Approved by the FDA for the treatment of erythromelalgia	A slight increase in body temperature in healthy individuals	^208,210^
			JYL1421	No	Blocked capsaicin and heat responses in human and monkey	Caused hypothermia in rats but hyperthermia in dogs and cynomolgus monkeys	^208,422^
		Agonists	Capsaicin	Yes	IA injection significantly alleviated KOA pain in a phase 2 trial	Evoked an intense initial pain reaction Rapid metabolism (with a half-life of 1.6 h) Occurrence of hypothermia, pruritus, erythema, and papules	^205,213,423^
			Resiniferatoxin	Yes	Significant relief of moderate to severe KOA pain in a phase 2a clinical trial	Postprocedural pain, nausea, vomiting, and headache in human trials	^421,424^
			CNTX-4975	Yes	IA injection significantly alleviated KOA pain in a phase 2 trial	Arthralgia, upper respiratory tract infection, increased hepatic enzymes, joint effusion, and osteoarthritis in human trials	^212^
			Zucapsaicin	Yes	Approved as a topical analgesic in 2010 for use in conjunction with oral COX-2 inhibitors or NSAIDs to relieve severe pain in adults with KOA	Stinging, burning, and erythema in human trials	^216^
			Olvanil (NE19550)	No	I.pl. injection alleviated capsaicin-induced thermal hyperalgesia in rats	Unknown	^205,425^
			MRD-652	No	Significant alleviation in the SNL model, formalin-induced, and capsaicin-induced allodynia model	Minimal signs of skin irritation and edema in the animal models	^216^
			CPIPC	No	P.o. alleviated CFA-induced inflammatory pain in mouse hind paws	Unknown	^213^
	TRPA1	Antagonists	HC-030031	No	P.o. reduced AITC-induced nocifensive behaviors, CFA-induced inflammatory pain, and the SNL model of neuropathic pain in rats without interfering with locomotor coordination or thermal sensitivity	No adverse effects reported	^426^
			AP-18	No	Reversed CFA-induced hyperalgesia in mice	Unknown	^427^
			A-967079	Yes	Systemic injection reduced mechanical stimulation in OA rats	Unknown	^428^
		Agonists	Atractylodin	No	Systemic injection reduced AITC-induced pain behaviors in rats	Moderate and prolonged nociceptive behaviors in rats	^221^
			Ms 9a-1	Yes	S.c. injection significantly reduced MIA-induced OA pain and inflammation in rats	Unknown	^429^
	TRPM8	Agonists	Menthol	Yes	The topical application of 3.5% menthol gel resulted in no significant pain relief in KOA patients	Caused cold pain, cold allodynia, and hyperalgesia at high concentration (30–40% wt/vol) in human trials	^228–230^
	TRPM3	Antagonists	Isosakuranetin	Yes	I.p. injection reduced ACLT-induced OA pain and progression in mice	Unknown	^430^
			Ononetin	No	Spinal administration prevented DMS-induced pain in rats	Unknown	^431^
			Primidone	No	Systemic administration attenuated PregS-induced pain in rats	Reduced motor exploratory activity in rats at high concentration (10 mg·kg^-1^)	^432^
Voltage-gated sodium channels	Pan-Na_v_ channels	Antagonists	Lidocaine	Yes	IA injection significantly attenuated KOA pain in a phase 2 clinical trial	Dizziness, sedation, convulsions, and cardiotoxicity were observed in human trials	^236,237,433^
			Mexiletine	No	Demonstrated no significant improvement in chronic pain in human trials	Mild neurological and gastrointestinal side effects in human trials	^434,435^
			Carbamazepine	Yes	Mitigated cartilage loss and reduced OA pain in mice	Transient drowsiness, loss of coordination, and vertigo in human trials	^436,437^
			Tricyclic antidepressants (TCAs)	Yes	Not clinically important for OA pain treatment	Nausea was reported as the most prevalent adverse event in human trials	^438^
	Na_v_1.7	Antagonists	PF-04856264	Yes	IA injection significantly alleviates OA pain and upregulates type II collagen levels in the murine meniscectomy model	No significant motor adverse effects were observed in mice	^245,439^
			ProTx II	Yes	Spinal administration attenuated MIA-induced OA pain in rats	Motor adverse effects at 3 m/kg were observed in a mouse model of acute postsurgical pain	^245–247,440^
			PTx2-3127	No	I.t. administration alleviated chronic and thermal pain in rats	No motor function decrements were observed in rats	^248^
	Na_v_1.8	Antagonists	A-803467	Yes	IA injection significantly alleviated hindlimb disability and secondary allodynia in MIA-induced OA rats	No adverse effect on motor function and motor coordination in rats	^247,249,441^
NGF	NGF	Antagonists	Tanezumab (RN624)	Yes	S.c. administration demonstrated greater OA pain relief than NSAIDs in a phase 3 clinical trial	Rapidly progressive OA, osteonecrosis, and fracture were observed in human trials	^251^
			Fasinumab (REGN475)	Yes	Significantly improved OA pain and physical function in a phase 3 clinical trial	Musculoskeletal and connective tissue disorders, nervous system disorders, injury, poisoning, and procedural complications were observed in human trials	^252,253,442^
			Fulranumab	Yes	Showed evidence in improving OA pain and physical function in a phase 3 clinical trial	No significant adverse effects in human trials	^443^
			ALE0540	No	I.p. and i.th. administration alleviated neuropathic pain and thermal-induced inflammatory pain in rats	Unknown	^254^
			PD90780	No	Not tested in pain models, but showed more inhibitory potential of NGF-TrkA binding than ALE0540 and Ro 08-2750	Unknown	^172,173,444^
			Ro 08-2750	No	Not tested in pain models	Unknown	^172,173^
			Y1036	No	Not tested in pain models	Unknown	^445^
			PQC 083	No	Not tested in pain models	Unknown	^444^
	TrkA	Antagonists	PF-06273340	No	Demonstrated efficacy in the UVIH model of inflammatory pain in rats	No adverse effects observed in rats	^255^
			AR786	Yes	P.o. significantly alleviated pain behaviors in MIA-induced OA rats	No significant adverse effects were detected in rats	^257^
CGRP	CGRP receptors	Antagonists	BIBN4096BS	Yes	S.c. Injection in MIA-induced OA rats provided analgesia similar to morphine within 4 h	No adverse effects were reported in rats	^259^
			CGRP 8-37	Yes	Reversed mechanosensitization of joint nociceptors in both MIA-induced and meniscectomy OA rats	Unknown	^260^
Glutamate-iGluRs	Pan-NMDA receptors	Antagonists	Ketamine	Yes	IA injection alleviated MIA-induced OA pain in rats	Psychotomimetic and cognitive adverse effects, and urological and hepatic toxicity for chronic application were observed in human trials	^446,447^
			Memantine	Yes	IA injection exhibited protective effects against cartilage degradation and partially reduced pain behaviors in MIA-induced OA rats	Unknown	^448^
			MK-801	Yes	Demonstrated anti-inflammatory efficacy in an OA cell model	Unknown	^449^
	NR2B-containing NMDA receptors	Antagonists	Ifenprodil	No	Spinal administration alleviated mechanical allodynia and neuropathic pain in rats	No specific prominent behavioral or psychological side effects were observed in human trials	^265,266,450^
			CP101606	No	P.o. administration alleviated carrageenan-induced hyperalgesia in rats	No motor impairment was observed in rats	^451^
			Ro256981	No	I.t. injection alleviated neuropathic pain in rats	Reduced memory in early life stress mice	^265,450^
			CI1041	No	Not tested in pain models	Unknown	^266^
	Pan-AMPA receptors	Antagonists	NBQX	Yes	IA injection reduced pain-related behaviors within 2 days in AIA rats but showed no analgesic effects in posttraumatic OA rats	Causes ataxia at doses above 40 mg·kg^-1^ in rats	^273,274,452^
	GluK1	Antagonists	MSVIII-19	No	I.t. administration reduced inflammatory pain and neuropathic pain in mice	No adverse effects on locomotor behavior in mice	^275^
			LY467711	No	P.o. reduced formalin-induced pain, carrageenan-induced thermal hyperalgesia, and capsaicin-induced mechanical hyperalgesia in rats	Unknown	^279^
			LY525327	No	P.o. reduced formalin-induced pain, carrageenan-induced thermal hyperalgesia, and capsaicin-induced mechanical hyperalgesia in rats	Unknown	^279^
Glutamate-mGluRs	Group I mGluRs	Antagonists	LY367385	No	Spinal administration reversed thermal hyperalgesia in rats	Unknown	^283^
			AIDA	No	Spinal administration reversed thermal hyperalgesia in rats	Unknown	^283^
	Group II mGluRs	Agonists	APDC	No	Blocked nociceptive hyperexcitability without affecting basal membrane excitability in human sensory neurons	Unknown	^281^
			LY379268	No	Reversed carrageenan-induced thermal hyperalgesia and capsaicin-induced mechanical allodynia in rats	Unknown	^280^
			LY389795	No	Reversed carrageenan-induced thermal hyperalgesia and capsaicin-induced mechanical allodynia in rats	Unknown	^280^
			LY354740	No	Reduced the pain behaviors in the formalin model of persistent pain in rats	Unknown	^280^
			LCCG1	No	Reversed capsaicin-induced mechanical allodynia in rats	Unknown	^280^
	Group III mGluR agonists	Agonists	ACPT-I	Yes	I.t. injection alleviated pain behaviors of OA rats	Unknown	^282^
			PHCCC	No	I.t. injection alleviated inflammatory and neuropathic pain in rats	Unknown	^282^

*KOA* knee osteoarthritis, *IA* Intra-articular, *COX-2* cyclooxygenase-2, *NSAIDs* nonsteroidal anti-inflammatory drugs, *i.pl*. intraplantar, *p.o*. oral administration, *SNL* spinal nerve ligation, *CFA* Complete Freund’s Adjuvant, *AITC* allyl isothiocyanate, *s.c*. subcutaneous, *i.p*. Intraperitoneal, *PregS* pregnenolone sulfate, *MIA* monosodium iodoacetate, *i.t*. Intrathecal, *UVIH* UV irradiation-induced thermal hyperalgesia

#### Dysfunctional organelle pharmacotherapy in osteoarthritis

Chondrocyte organelles function as a complex, compartmentalized network essential for normal cell operations. In OA, pathological changes in these organelles are closely linked to chondrocyte differentiation, apoptosis, and cartilage degeneration. Repairing dysfunctional organelles is a promising therapeutic strategy to restore chondrocyte function and support cartilage repair. Targeting organelle-related processes involved in OA progression may help reestablish the cellular balance and maintain cartilage integrity, suggesting viable strategies for future clinical OA treatment (Table 2).

**Table 2:** Summary of potential anti-inflammatory agents for OA treatment

Targets	Genre	Drug	Tested in OA	Preclinical or clinical effects	Adverse effects	Ref.
IL-1	IL-1RI Antagonists	Anakinra	Yes	IA injection significantly alleviated OA pain and improved WOMAC scores in clinical trials	Injection-site effects, anaphylaxis, pneumonia, and cellulitis in clinical trials	^56,64,291^
		AMG 108	Yes	I.v. and s.c. administration significantly reduced CRP and neutrophil counts in OA patients but failed to improve the OA condition	No significant AEs were observed in clinical trials	^293^
		Canakinumab	Yes	Significant OA improvements in clinical trials	Serious infections in clinical trials	^453^
	IL-1-targeting agents	Rilonacept	No	Demonstrated sustained improvements in systemic and joint symptoms in >50% JIA patients over 2 years	Injection-site reactions and infections (most commonly reported were URTIs and viral gastroenteritis) in clinical trials	^454^
		Rilabnacept	No	Not tested in OA models	Unknown	^291^
		Bermekimab	No	Not tested in OA models	Infections (cellulitis, influenza virus infection, upper respiratory infection, urinary tract infection) in clinical trials	^455^
		Gevokizumab	No	Not tested in OA models	Infections (conjunctivitis, URTI) in clinical trials	^455^
		Lutikizumab	Yes	Adequate blockade of IL-1, but failed to achieve improvements in erosive HOA	Injection-site reactions and neutropenia in clinical trials	^456^
	Caspase-1 inhibitors	VX-740	Yes	Significantly reduced KOA progression in mice	Liver toxicity in animal studies	^295^
		VX-765	Yes	Reduced the inflammatory responses and improved the damage to the joint structure in OA rats	Unknown	^296^
TNF-α	TNF-α-targeting agents	Infliximab	Yes	IA injection significantly reduced the TNF-α levels in the synovial fluid and the pathological changes of the joint in OA rabbits IA injection insignificantly reduced HOA progression in a clinical trial	No local or systemic adverse reactions were reported in clinical trials	^457,458^
		Etanercept	Yes	S.c. administration insignificantly reduced HOA progression in a clinical trial	Infections in clinical trials	^459^
		Adalimumab	Yes	Marked improvement in KOA pain intensity and quality of life No improvements for HOA in clinical trials	No significant adverse effects were reported in clinical trials	^460^
		Certolizumab pegol	No	Rapid improvements for moderate-severe RA patients	Infections in clinical trials	^461^
	TNFR-1 antagonists	PEGylated r1antTNF	No	I.v. injection significantly reduced the development and severity of CIA in a mouse model	No adverse effects were reported	^307^
		Atrosimab	No	I.v. injection significantly reduced the development and severity of arthritis in a mouse model	No adverse effects were reported	^308^
		IZI-06.1	No	Not tested in OA models	Unknown	^306^
		TNFR1-AlbudAb	No	Not tested in OA models	Unknown	^306^
		Nb 70	No	Not tested in OA models	Unknown	^462^
		Nb 70	No	Not tested in OA models	Unknown	^462^
		PMG	No	Not tested in OA models	Unknown	^463^
		R1	No	Not tested in OA models	Unknown	^464^
	Noncompetitive TNFR-1 antagonists	DS41	No	Not tested in OA models	Unknown	^306^
		DSA11	No	Not tested in OA models	Unknown	^306^
		Zafirlukast	Yes	Exerted potent anti-osteoarthritic effects in human primary chondrocytes	Unknown	^465^
IL-6	IL-6-targeting agents	Tocilizumab	Yes	No significant improvements in HOA patients	Infections and neutropenia were reported in clinical trials	^317^
		Olokizumab	No	S.c. injection demonstrated significant improvements in RA patients	Infections were reported in clinical trials	^466^
		Siltuximab	No	Relieved all symptoms of sJIA patients	Thrombocytopenia, hypertriglyceridemia, neutropenia, leukopenia, hypercholesterolemia, and anemia were reported in clinical trials	^467^
		Sirukumab	No	S.c. injection significantly reduced symptoms in RA patients	Elevated liver enzymes, URTI, injection site erythema, and nasopharyngitis were reported in clinical trials	^468^
		Clazakizumab	No	S.c. injection demonstrated significant improvements in RA patients	Appendicitis, cellulitis, Pneumonia, and Pulmonary tuberculosis were reported in clinical trials	^469^
		Olamkicept	No	Inhibited IL–6–mediated TH17 cell expansion in disease models of RA	upper respiratory infections, recurrence of herpes labialis, and skin and s.c. disorders were reported in clinical trials	^318^
		Cs-130Fc	No	Not tested in OA models	Unknown	^318^
	IL-6R antagonists	Sarilumab	No	S.c. injection demonstrated significant improvements in RA patients	Infections and neutropenia were reported in clinical trials	^470^
		Satralizumab	No	Not tested in OA models	Urinary tract infections	^471^
		Vobarilizumab	No	S.c. injection demonstrated significant improvements in RA patients	No significant adverse effects were reported	^472^
	Gp130 antagonists	SC144	Yes	Significantly reduced the basal release of NO and IL-6 with no effect on GAG production and cytotoxicity in KOA chondrocytes	Unknown	^315^
	Pan-JAK antagonists	Tofacitinib	Yes	IA injection significantly reduced arthritis scores and bone degradation in an OA mouse model	Unknown	^473^
		Baricitinib	Yes	Demonstrated significant improvements in OA patients	URTI	^474^
	JAK-1 antagonists	Filgotinib	No	Demonstrated significant improvements in RA patients	Nasopharyngitis, URTI, nausea, urinary tract infection	^475^
	TYK2 antagonists	Deucravacitinib	No	P.o. demonstrated significant improvements in PsA patients	Nasopharyngitis, head ache, diarrhea, nausea, and upper respiratory tract infection	^476,477^
	STAT3 antagonists	OPB-31121	No	Not tested in OA models	Unknown	^310^
		OPB-51602	No	Not tested in OA models	Unknown	^310^

*WOMAC* Western Ontario and McMaster Universities Arthritis Index, *i.v*. intravenous, *s.c*. subcutaneous, *CRP* C-reactive protein, *AE* adverse effect, *JIA* juvenile idiopathic arthritis, *HOA* hand osteoarthritis, *KOA* knee osteoarthritis, *IA* Intra-articular, *RA* rheumatoid arthritis, *CI* collagen-induced arthritis, *sJIA* Systemic-Onset juvenile idiopathic arthritis, *GAG* glycosaminoglycan, *URTI* Upper respiratory tract infection, *p.o*. oral administration, *PsA* psoriatic arthritis

##### Mitochondrial pharmacotherapy in osteoarthritis

Mitochondrial homeostasis is crucial for chondrocytes to generate sufficient cellular energy and maintain normal cellular function. However, studies have shown that mitochondrial biogenesis-related proteins, such as AMPK, SIRT1, PGC-1α, NRF1, NRF2, and TFAM, are downregulated in OA chondrocytes from both humans and mice.^97,322^ In addition, decreased expression of mitochondrial respiratory complexes I–IV and ATP synthase (Complex V) results in insufficient energy production.^323^ The buildup of dysfunctional mitochondria also leads to excessive ROS production, contributing to chondrocyte damage and apoptosis.^101,324^ Therefore, restoring mitochondrial biogenesis by upregulating biogenesis-related proteins or enhancing mitophagy through proteins such as PINK1, Parkin, TOM, and LC3 to remove damaged mitochondria is a promising therapeutic strategy for OA. Potential drugs targeting these pathways, categorized by their roles in mitochondrial biogenesis or mitophagy, are summarized in Table 3.

**Table 3:** Potential therapeutic agents for restoring organelle dysfunction in OA

Organelles	Therapeutic agents	Therapeutic target	Mechanism	Activity	Disadvantages	Ref.
Mitochondria	Quercetin	Mitochondrial biogenesis	AMPK/SIRT1/PGC-1α pathway	Enhanced expression of AMPK, SIRT1, PGC-1α, NIRF1, NIRF2 and TFAM (murine meniscectomy model)	Unknown	^478^
	Fibroblast growth factor 19	Mitochondrial biogenesis	AMPK/SIRT1/PGC-1α pathway	Enhanced expression of AMPK PGC-1α and SIRT1 (murine chondrocytes)	Potential risk of metabolic disorders	^479^
	Omentin-1	Mitochondrial biogenesis	PGC-1α/TFAM pathway	Enhanced expression of PGC-1α, NRF1 and TFAM	Unknown	^98^
	Catapol	Mitochondrial biogenesis	PGC-1α/TFAM pathway	Enhanced expression of PGC-1α, NRF1 and TFAM (murine ATDC5 chondrocytes)	Lack of in vivo studies	^480^
	Sestrin 2	Mitochondrial biogenesis	AMPK/PGC-1α pathway	Enhanced expression of mtDNA copy number, PGC-1α, NIRF1, NIRF2 and TFAM (murine MIA-induced OA model)	Unknown	^96^
	Urolithin A	Mitophagy	PINK1/Parkin pathway	Upregulation of mitophagy-related proteins encoded genes and increased level of ph-Ub (human chondrocytes)	Limited repair ability of chondrocytes	^481^
	Dihydromyricetin	Mitophagy	PINK1/Parkin pathway	Activation of PINK1/Parkin-LC3B axis (murine chondrocytes)	Unknown	^482^
	Mitochonic acid-5	Mitophagy	PINK1/Parkin pathway	ΔΨm Restoration, enhanced expression of LC3B, Parkin (Human OA chondrocytes)	Lack of in vivo studies	^483^
	α-ketoglutarate	Mitophagy	PINK1/Parkin pathway	Enhanced expression of PINK1, Parkin, TOMM7 (murine ACLT model)	Low oral bioavailability	^484^
	17b-estradiol	Mitophagy	SIRT1/AMPK/mTOR pathway	Enhanced expression of SIRT1, LC3 and TOM20 (murine ATDC5 chondrocytes)	Unknown	^485^
Lysosome	Astaxanthin	Hydroxyapatite formation	Inhibited expression of RUNX2	Inhibition of hypertrophic chondrocyte differentiation (murine TBHP-induced IVDD model)	Inconclusive effect in OA	^486^
	Adseverin	Hydroxyapatite formation	Inhibited expression of RUNX2 and type X collagen	Inhibition of hypertrophic chondrocyte differentiation (murine meniscectomy model)	Unknown	^487^
	Melatonin	Hydroxyapatite formation	Inhibited expression of RUNX2	Inhibition of chondrocyte mineralization (murine TBHP-induced chondrocytes)	Lack of in vivo studies	^488^
	CD11b agonists	Hydroxyapatite formation	May inhibit the expression of ALP and ANX V	May inhibit chondrocyte mineralization	Inconclusive results in OA	^130^
	Krüppel-like factor 10 inhibitors	Hydroxyapatite formation	Inhibited gene expression of RUNX2	Inhibition of chondrocyte mineralization (murine TBHP-induced chondrocytes)	No formulation available	^489^
	lymphoid enhancer-binding factor 1 (Lef1) inhibitors	Hydroxyapatite formation	Inhibited expression of RUNX2	Inhibition of chondrocyte mineralization (murine Lef1 knock-out costal chondrocytes)	No formulation available	^490^
	Synovium-derived stromal cells	Hydroxyapatite formation	Inhibited expression of RUNX2 and type X collagen	Inhibition of hypertrophic chondrocyte differentiation (murine costal chondrocytes)	No formulation available	^491^
ER	4-PBA	ER stress	Assist protein folding	Inhibited expression of BiP, MMP-13, ADAMTS 5, and CHOP (murine meniscectomy model)	Unknown	^340^
	TUDCA	ER stress	Assist protein folding	Inhibited expression of BiP and increased expression of type II collagen (human OA chondrocytes)	lack of in vivo studies	^339,341^
	MG-132	ER stress	Assist protein folding	Alleviation of chondrocyte apoptosis and cartilage degradation (murine MIA-induced OA model)	Unknown	^339,492^
	HSP 70	ER stress	Assist protein folding	Inhibition of expression of caspase 3 and restoring the biosynthesis of proteoglycan (murine MIA-induced OA model)	No adverse effects were reported	^493^
	HSP 90 A	ER stress	Assist protein folding	Alleviation of chondrocyte apoptosis (human OA chondrocytes)	No formulation available	^494^
	Salicin	ER stress	Inhibition of IRE1α phosphorylation by binding to the phosphorylation site of IRE1α	Inhibited expression of IRE1α-mediated apoptotic genes (IκBα, p65) (murine ACLT model)	No adverse effects were reported	^495^
	Vitexin	ER stress	Unknown	Inhibited expression of CHOP (murine TG-induced chondrocytes with ER stress)	Unknown	^496^
	Safranal	ER stress	Inhibition of PERK-ATF4-eIF2a signaling pathway via the activation of SIRT1	Inhibited expression of CHOP (murine meniscectomy model)	Unknown	^497^
	Trehalose	ER stress	Unknown	Inhibited expression of BiP and CHOP (murine TBHP-induced chondrocytes)	lack of in vivo studies	^498^
	Dapagliflozin	ER stress	Inhibition of PERK-ATF4-eIF2a signaling pathway via the activation of SIRT1	Inhibited expression of BiP, PERK, eIF2α, ATF4 and CHOP (murine IL-1β-induced chondrocytes)	Unknown	^499^

*MIA* monosodium iodoacetate, *ACLT* anterior cruciate ligament transection, *TBHP* tert-butyl hydroperoxide, *IVDD* intervertebral disc degeneration, *4-PBA* 4-phenylbutyric acid, *TUDCA* tauroursodeoxycholic acid, *HSP 90A* heat shock protein 90A, *MIA* monosodium iodoacetate, *TG* triglyceride

##### Lysosomal pharmacotherapy in osteoarthritis

Lysosomes play a vital role in degrading cellular waste and damaged organelles through hydrolytic enzymes, thereby supporting ECM homeostasis. Lysosomal destabilization, which triggers chondrocyte apoptosis, is implicated in the pathogenesis of OA. A study conducted by Tao et al. revealed that the phagocytosis of hydroxyapatite crystallites by chondrocytes can disrupt lysosomal integrity.^113^ Consequently, therapeutic agents capable of mitigating cartilage calcification may represent promising drugs for slowing OA progression by stabilizing lysosomes. Ideal candidates would inhibit chondrocyte hypertrophic differentiation or interfere with Ca²⁺ and P_i_ binding during cartilage calcification without causing severe side effects.

Over the past few years, several therapies aimed at reducing cartilage calcification have been explored at the cellular and animal levels, offering potential treatments for OA. Recent developments in this area are summarized in Table 3. These therapies help maintain the chondrocyte phenotype by downregulating the expression of calcification-promoting genes and proteins, such as RUNX2 and type X collagen, or by reducing ACP formation in MVs, which may prevent crystal-induced LMP. However, caution is warranted, as some of these therapies have not been tested in OA models, and no relevant animal experiments have been conducted.

Furthermore, the use of cathepsin inhibitors to preserve BID integrity, along with functional molecules that alleviate LMP—such as heat shock protein 70 (Hsp70), annexin A7, endosomal sorting complex required for transport protein III (ESCRT-III), LAMP-1, and LAMP-2—has been proposed as an alternative treatment to prevent lysosome-induced chondrocyte apoptosis.^325,326^ Current studies suggest that cathepsins B and D primarily cleave BID. Specific inhibitors have been developed for cathepsins B (such as CA-074 and CA-030) and D (e.g., pepstatin A), which are used to treat various diseases.^327,328^ However, the efficacy of these inhibitors in reducing lysosome-induced chondrocyte apoptosis remains unconfirmed, as no published studies have specifically addressed this issue. Notably, inhibitors of cathepsins K and L have been widely studied for OA treatment because of the high expression of these enzymes in osteoclasts, where they are responsible for collagen degradation in bone and cartilage.^329,330^ Additionally, Hsp70 is an upstream regulator of ASM involved in the normal metabolism of SM.^331,332^ Annexin A7 and ESCRT-III are components of the plasma membrane repair system that facilitate the resealing of damaged lysosomal membranes by aggregating at the injury site.^326,332^ Moreover, LAMP-1 and LAMP-2 constitute ~50% of lysosomal membrane proteins and are essential for maintaining lysosomal morphology.^333^ Thus, the exogenous administration of recombinant forms of these proteins or their agonists may help repair hydroxyapatite-induced LMP in chondrocytes. This approach is supported by a study showing that recombinant Hsp70 (rHsp70), combined with the Hsp70 inducer arimoclomol, corrected pathological lysosomal morphology in primary fibroblasts from patients with Niemann–Pick disease.^334^

##### Endoplasmic reticulum pharmacotherapy in osteoarthritis

The ER is a central organelle for the proper synthesis, maturation, and folding of proteins. Over one-third of proteins in eukaryotic cells are folded within the ER lumen.^138^ This substantial workload makes the ER highly susceptible to environmental stimuli, often leading to errors and the accumulation of misfolded proteins, which induce ER stress. The UPR signaling pathway, which is activated in response to ER stress, helps restore ER homeostasis to some extent. However, if stress becomes irreversible, it triggers apoptosis. Upon elaboration of ER stress-associated mechanisms, two therapeutic strategies are under investigation for pharmacological intervention: one focuses on reducing or eliminating the aggregation of misfolded proteins in the ER lumen, whereas the other targets the modulation of stress sensor activity (PERK, IRE1α, and ATF6α) or the enzymes involved in the UPR.^335^

PERK and IRE1α are known to initiate apoptotic processes in cells. Emerging studies have tested inhibitors of these sensors in various ER stress-related diseases, including cancer, neurodegenerative disorders, and obesity. GSK2656157, a PERK inhibitor, effectively reduces cancer growth but is discontinued because of its toxicity.^336^ ISRIB, a small molecule that inhibits eIF2α phosphorylation, thereby reducing CHOP expression, has been found to enhance cognitive memory in brain-injured mice without severe side effects.^337^ Inhibitors of IRE1α, such as 4µ8c, MKC3946, and STF-083010, reversed hepatic steatosis in animal models.^335^ While these agents may help alleviate ER stress during OA progression, their efficacy in OA models remains untested.

Another therapeutic strategy for treating ER stress involves reducing the aggregation of misfolded proteins. Certain small molecules and proteins can alleviate this aggregation in the ER, offering potential benefits for OA. Chemical chaperones are small molecular compounds that reduce the free movement of proteins, which helps prevent the aggregation of misfolded proteins. They also increase the free energy between misfolded and native proteins, promoting proper folding to restore a more stable state.^338^ However, chemical chaperones are nonselective and often require high concentrations to be effective, which may cause toxicity and limit their application in vivo.^335,338,339^ Nonetheless, two chemical chaperones, 4-phenylbutyric acid (4-PBA) and tauroursodeoxycholic acid (TUDCA), are FDA-approved for human use.^339^ Among these, 4-PBA reduced cartilage destruction in a murine meniscectomy model, whereas TUDCA decreased the expression of ER stress-related markers and increased type II collagen expression.^340,341^

Pharmacological chaperones (PCs) are another type of small molecule designed to selectively bind to misfolded proteins and bridge noncovalent interactions lost or loosened during misfolding, thereby promoting proper folding.^342,343^ Compared with chemical chaperones, PCs work at lower concentrations and are more selective, making them promising therapeutic agents for OA. Cellular molecular chaperones are proteins that prevent the aggregation of misfolded proteins by recognizing and binding to their hydrophobic regions.^344^ The most common group of molecular chaperones is heat shock proteins (Hsps), which are classified by their molecular weight into Hsp40s, Hsp60s, Hsp70s, Hsp90s, Hsp100s, and small Hsps. These proteins are also involved in macromolecular complex assembly, protein transport, degradation, and the refolding of misfolded proteins,^345^ making them promising candidates for treating ER stress. Some molecular chaperones tested in OA chondrocytes are summarized in Table 3.

Certain chemical compounds and natural products derived from plants, fruits, and bacteria significantly downregulate the expression of ER stress-induced apoptotic genes or proteins (such as BiP, IRE1α, PERK, eIF2α, ATF4, and CHOP), positioning them as potential candidates for OA treatment (Table 3). However, the exact mechanisms through which these compounds alleviate ER stress are not fully understood, and further research is needed to elucidate how they affect ER stress pathogenesis.

### Organelle-targeted drug delivery systems

Pharmacological molecules that target chondrocyte organelles are often limited by membrane barriers, considerably reducing their therapeutic efficacy. To maximize the effectiveness of these molecules, additional pharmacological modifications are necessary to increase their absorption by chondrocytes and organelles, improve their cellular distribution, and minimize toxicity. Various cell-penetrating peptides and cell-penetrating poly(disulfide)s are commonly used for their ability to promote cellular uptake and facilitate efficient intracellular delivery of therapeutic cargos, including small molecules, plasmid DNA, siRNA, therapeutic proteins, viruses, imaging agents, and other nanomedicines.^346^ However, they are commonly modified on the surface of nanomedicines (nanoparticle, liposome, exosome, polymeric micelles) along with other functional ligands instead of being directly conjugated with pharmacological molecules due to a lack of cell/tissue specificity.^347,348^ These synthetic pharmacological formulations, referred to as drug delivery systems (DDSs), are designed to improve the physicochemical properties, pharmacokinetics, and organelle-targeting capabilities of pharmacological molecules. Proper DDS design can enhance the functionality of chondrocyte organelles, offering potential improvements in OA treatment.

#### Mitochondria-targeting

**Fig. 6:**
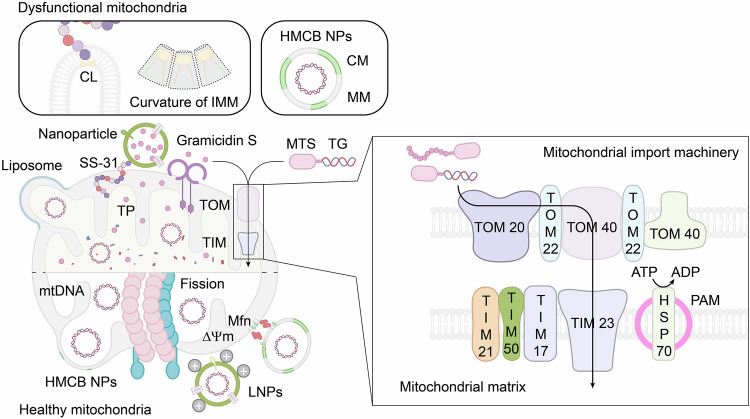
Schematic representation of advanced therapeutic strategies for mitochondrial targeting in chondrocytes to address mitochondrial dysfunction in OA. The diagram highlights two principal strategies: the delivery of mtDNA, therapeutic proteins, and therapeutic genes to dysfunctional mitochondria to foster repair and the delivery of healthy mtDNA to healthy mitochondria to increase biogenesis. The figure emphasizes the application of liposomes and hybrid membrane-coated biomimetic nanoparticles as cutting-edge delivery systems for healthy mitochondria, leveraging the ΔΨm for binding. For dysfunctional mitochondria in OA, where the ΔΨm is compromised, potential-independent strategies such as SS-31 and gramicidin S derivatives are employed for targeting. The mitochondrial import machinery, including the TOM and TIM complexes, is crucial for the translocation of therapeutic agents into the mitochondrial matrix. TOM facilitates the initial import of nuclear-encoded proteins into the mitochondrial matrix, whereas TIM assists in their further translocation across the inner mitochondrial membrane, aided by mtHsp70, which utilizes ATP hydrolysis to drive the process. HMCB NPs hybrid membrane-coated biomimetic nanoparticles, LNPs lipophilic nanoparticles, TP therapeutic protein, TG therapeutic gene, CM chondrocyte membrane, MM mitochondrial membrane, CL cardiolipin, TOM translocase of the outer mitochondrial membrane, TIM translocase of the inner mitochondrial membrane, mtDNA mitochondrial DNA, ΔΨm mitochondrial membrane potential, mtHsp70 mitochondrial chaperone heat shock protein 70

In OA, ROS and an inflammatory environment damage the normal functions of mtDNA, leading to an increased number of dysfunctional mitochondria. This dysfunction contributes to chondrocyte lysis and cartilage degradation, making the repair or elimination of dysfunctional mitochondria through mtDNA rehabilitation a promising approach for OA treatment. Current strategies to repair damaged mtDNA involve delivering healthy mtDNA into the matrices of dysfunctional mitochondria; introducing functional molecules to repair, degrade, or inhibit the replication of damaged mtDNA; or promoting the replication of healthy mtDNA in normal mitochondria to increase mitochondrial biogenesis.^349^ However, owing to the nonselective distribution of therapeutic molecules in vivo and the unique structure of mitochondria—with their hydrophobic, dense double membrane, stringent transport machinery, and negative potential (approximately −160 to −180 mV) across the IMM—delivering drugs to mitochondria remains challenging. Therefore, additional carriers are needed to overcome these barriers.^350,351^ Strategies targeting mitochondria are discussed below (Fig. 6).

##### Lipophilic cations

Mitochondria-targeting carriers have been extensively studied in recent years, and most of them share the common trait of possessing lipophilic and cationic properties that enable the selective targeting of normal mitochondria through electrostatic interactions.^352^ Lipophilic cations such as triphenylphosphonium (TPP)-based cations, rhodamine 123 (Rh123), cyanine cations, and cationic peptides are usually conjugated synergistically with other functional ligands on the surface of nanomedicines such as nanoparticles, liposomes, and vesicles, in which therapeutic molecules are encapsulated. This approach enhances tissue/cell specificity while increasing their permeability and bioavailability, regulating their distributions in vivo, and managing their release rates with minimal side effects.^352–354^ For example, Huang et al. engineered a PEG/TPP-modified nanoparticle loaded with a natural anti-inflammatory compound, rhein, for delivery to mitochondria in IL-1β-stimulated chondrocytes. These nanoparticles exhibited anti-inflammatory effects by inhibiting IL-1β, IL-6, TNF-α, and MMP-13; upregulated the expression of anabolic genes (Col2a1 and Acan); protected the ΔΨm; and inhibited chondrocyte apoptosis.^355^ Yu et al. developed the pH-sensitive and TPP-modified nanoparticle DOX-PLGA/CPT/PD on the basis of the acidic property of the tumor environment for the delivery of DOX to mitochondria in tumor cells. The DDS is composed of positively charged nanoparticles synthesized from poly-lactide-co-glycolic acid (PLGA) and a TPP-containing amphiphilic polymer, with a negatively charged PD shell on the outer layer. This formulation ensures electroneutrality at physiological pH, preventing rapid clearance and nonselective ionic interactions in vivo. In an acidic tumor environment, the PD shell detaches from the nanoparticle, releasing the TPP-modified nanoparticle through amino bond breakage, resulting in improved DOX uptake and increased mitochondrial DOX concentrations in tumor cells.^350^ Similarly, Yuan et al. developed a biodegradable silica nanoparticle conjugated with TPP and CPD on the surface to deliver macromolecules to the mitochondria. The results further demonstrated the successful delivery of the native protein _FL_BSA and an anti-MTCO2 antibody to the mitochondria in HeLa cells.^356^ These strategies can also be adapted to deliver functional molecules, such as the mitochondrial outer membrane protein MDI (CG3249) and C17orf80, which promote mtDNA replication in the normal mitochondria of chondrocytes to treat OA.^357,358^ However, developing pharmaceutical formulations tailored specifically for OA is essential to overcome challenges such as the rapid in vivo clearance of cationic ligand-modified nanomedicines and the limitations of lipophilic cations, which primarily localize to the mitochondrial surface rather than the matrix. Furthermore, nonselective ionic interactions between cationic ligands and anionic proteins in the bloodstream may induce unintended side effects.^350,352^

##### Potential-independent mitochondria-targeting strategies

The promising mitochondria-targeting properties of lipophilic cations have spurred significant interest in exploring their therapeutic potential in various mitochondria-related diseases. However, these strategies are unlikely to effectively target depolarized mitochondria with impaired ΔΨm in chondrocytes.^353^ Therefore, potential independent mitochondria-targeting approaches may offer more suitable alternatives for treating OA.

Szeto-Schiller 31 (SS-31), a short-chain peptide consisting of ~10 amino acids, targets the IMM independently of ΔΨm through interaction with cardiolipin, a unique phospholipid residing on the IMM.^354,359^ Owing to its small size, ease of synthesis, water solubility, favorable safety profile (with injection site reactions as the predominant side effect and no fatal events reported), and relatively long half-life demonstrated in animal studies (~2 h in rats, dogs, and monkeys),^354^ SS-31 is an ideal candidate for targeting dysfunctional mitochondria in chondrocytes.

Furthermore, derivatives of gramicidin S, which are traditionally known as antibiotics because of their ability to disrupt bacterial membranes, are also being explored as mitochondria-targeting carriers because of the similarities between bacterial and mitochondrial membranes. XJB-5-131, a derivative of gramicidin S, has been shown to be inserted into the IMM and deliver a ROS scavenger (4-AT) without dependence on the ΔΨm, suggesting its potential for targeting depolarized mitochondria in chondrocytes.^354,360^ However, no mitochondrion-targeted pharmaceutical formulations using SS-31 or gramicidin S derivatives have been established for OA treatment, although relevant insights can be gleaned from other studies.

For example, Zhang et al. developed a PEG-PLGA nanoparticle conjugated with SS-31 that encapsulated cyclosporine A (CsA@PLGA-PEG-SS31) for treating myocardial ischemia‒reperfusion. The results demonstrated enhanced mitochondria-targeting efficacy and desirable cardioprotective effects.^359^ Similarly, Kuang et al. designed SS-31-conjugated geranylgeranylacetone-loaded PLGA nanoparticles that exhibited specific accumulation in mitochondria and enhanced ΔΨm in hair cells.^361^

Recently, Li et al. reported the development of mitochondrion-targeted polyethylene glycol-fluorocarbon (PEG_m_-F_n_) self-assembled micelles, which consist of a fluorinated core and a PEGylated shell. PEG_m_ balances the strong hydrophobicity of F_n_ to form stable micelles, which disengage upon reaching the cell membrane due to its “stealth property.” Once internalized, F_n_ acts as a drug carrier and facilitates mitochondrial accumulation through interaction with cardiolipin on the IMM. These micelles exhibit electrically neutral properties, effective cellular uptake, endosomal escape, and successful mitochondrial targeting across various cell types.^352^ This strategy addresses both the challenges of cell membrane penetration and mitochondria targeting, positioning PEG_m_-F_n_ self-assembled micelles as a promising approach for mitochondrial targeting in chondrocytes.

##### Strategies for accessing the mitochondrial matrix

Strategies for accessing the mitochondrial matrix involve the intricate process of navigating the mitochondrial import machinery. While lipophilic cations and potential-independent mitochondria-targeting carriers are effective at delivering therapeutic cargo to mitochondria, their ability to reach the mitochondrial matrix, where therapeutic effects are exerted, is often limited. To overcome this limitation, modification of the therapeutic cargo is necessary.

Substances entering the mitochondria are tightly regulated by the mitochondrial import machinery, which includes various translocases, such as the TOM and translocase of the inner membrane (TIM) complexes.^354^ The TOM complex, an oligomeric structure equipped with multiple channels, is responsible for recognizing and translocating proteins across the OMM. In this process, TOM40, in collaboration with TOM20, which identifies protein presequences, catalyzes the initial import of proteins into the intermembrane space. TOM20, characterized by its ability to adopt multiple conformations and interaction modes, specifically recognizes the presequences of proteins destined for the mitochondrial matrix. Upon recognition, TOM20 facilitates the translocation of these proteins to the central receptor TOM22, thereby initiating their import into the intermembrane space.

Conversely, the translocation of proteins destined for the inner mitochondrial membrane is orchestrated by the TIM complex, which plays a crucial role in their passage across this barrier. The TIM23 complex, which serves as the channel core of the TIM machinery and is composed of TIM17, TIM21, and TIM50, is complemented by the motor protein PAM. This motor protein is encircled by the mitochondrial chaperone heat shock protein 70 (mtHsp70), which is instrumental in catalyzing ATP hydrolysis. This ATP hydrolysis powers the translocation of proteins into the mitochondrial matrix. The synergistic function of the TOM and TIM complexes is indispensable for the precise and efficient delivery of therapeutic agents to the mitochondrial matrix, ensuring the accurate transport of proteins to their designated locations within the mitochondria.^354,362,363^

Most mitochondrial-targeted proteins possess an MTS, which is essential for their transport to the matrix. Over half of the MTSs are localized at the N-terminus of proteins and often adopt an amphiphilic helical structure, with one positively charged surface and one hydrophobic surface—features believed to be necessary for matrix translocation.^353,364^ Conjugating MTSs with therapeutic genes or proteins in DDSs can facilitate their delivery to the mitochondrial matrix. However, proteins transported to the matrix by MTSs must typically be unfolded, which may compromise their therapeutic efficacy to some extent.^354^

##### Other mitochondrial-targeting strategies for osteoarthritis treatment

The size and complexity of DDSs often limit the efficiency of therapeutic cargo delivery to mitochondria.^349,354^ To address this issue, several innovative strategies have been proposed. Yamada et al. developed a mitochondrial fusogenic liposome (termed the MITO-Porter DDS) on the basis of the concept that mitochondria undergo fusion and fission to share biomolecules. This system directly delivers the encapsulated therapeutic cargo to mitochondria by fusing with the mitochondrial membrane, regardless of the physical properties of the cargo,^349^ making the MITO-Porter DDS a promising candidate for gene or protein delivery in OA treatment.

In recent years, biomimetic nanoparticles with hybrid membranes from cancer cells and mitochondria that encapsulate therapeutic molecules have been developed for cancer treatment by leveraging homotypic binding between cancer and mitochondrial membranes.^365^ Membrane-camouflaged nanoparticles combine the physicochemical benefits of synthetic materials with their biological features, enabling efficient mitochondrial delivery of therapeutic molecules by modulating the ΔΨm in target cells.^366^ A similar approach could be adapted for OA treatment. Deng et al. developed chondrocyte membrane-coated nanoparticles for the treatment of cartilage damage. They demonstrated that chondrocyte membranes exhibit inherent binding affinity to the ECM, promoting longer drug retention and facilitating homotypic binding between chondrocytes, which enhances nanoparticle internalization.^367^ These findings provide both theoretical and practical foundations for the development of biomimetic nanoparticles for chondrocyte mitochondrial targeting in OA treatment.

#### Lysosome targeting

**Fig. 7:**
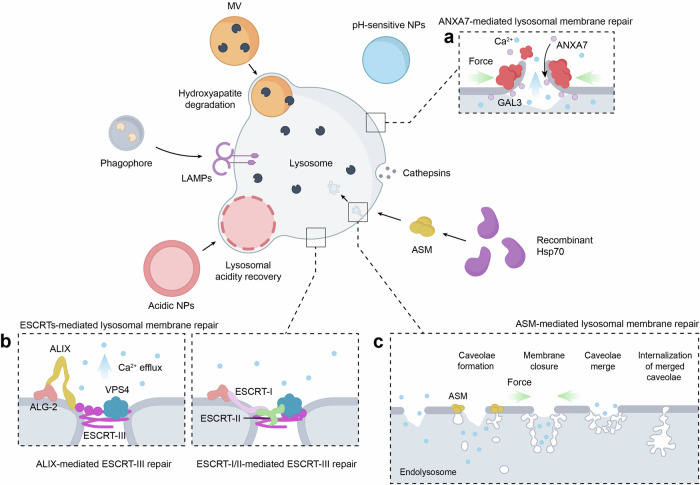
Schematic representation of dual therapeutic approaches for lysosomal dysfunction in OA, with a focus on the restoration of lysosomal acidity and the repair of lysosomal membranes. The restoration of lysosomal acidity is facilitated by the delivery of acidic nanoparticles designed to release their contents within the acidic lysosomal environment, thereby enhancing the degradation of hydroxyapatite crystallites. Concurrently, the delivery of functional molecules such as Hsp70 alleviates LMP and promotes lysosomal membrane repair. The repair mechanisms involve three distinct pathways: **a** ANXA7-mediated repair, where ANXA7 is recruited to the lysosomal membrane upon calcium influx. **b** ESCRT-mediated repair, involving the activation of ESCRT-III components and Ca²⁺-binding protein ALIX or ESCRT-I/II pathways in response to endolysosomal membrane damage, leading to the formation of a higher-order structure that closes the membrane breach by ATPase VPS4. **c** ASM-mediated repair, where ASM activity induces caveolae formation, followed by membrane closure and internalization of merged caveolae, thus restoring lysosomal membrane integrity. These strategies are crucial for mitigating lysosome-induced chondrocyte apoptosis and are highlighted as necessary for lysosome-targeting strategies in OA therapy. MV matrix vesicle, ASM acid sphingomyelinase, Hsp70 heat shock protein 70, LMP lysosomal membrane permeabilization, LAMPs lysosome-associated membrane proteins

The direct elimination of excess hydroxyapatite crystallites within dysfunctional lysosomes—either by restoring lysosomal acidity to enable degradability or by introducing crystal-degrading agents—is a critical component of OA treatment. Targeting palliative functional molecules that alleviate LMP in dysfunctional lysosomes further underscores the importance of lysosome-targeting strategies in OA therapy. One advantage of designing lysosome-targeting DDSs is that most naturally follow the endosome‒lysosome trafficking route,^368^ eliminating the need for additional lysosomal-targetable moieties. Furthermore, pH-sensitive DDSs that remain stable at physiological pH but release their encapsulated cargo in acidic environments, such as the lysosomal lumen (pH 4.5–5.0), are commonly utilized to increase lysosome targetability (Fig. 7).^369,370^

With respect to hydroxyapatite crystallite elimination, no crystal-degrading agents other than hydrochloric acid, which directly degrade hydroxyapatite, have been reported to date.^115,371^ However, several studies have successfully restored lysosomal acidity. For example, Zeng et al. developed pH-sensitive polyester-coated nanoparticles (acNPs) encapsulating fluorinated diacid and tetrafluorosuccinic acid for nonalcoholic fatty liver disease treatment. The restoration of dysfunctional lysosomal acidification, improved mitochondrial function, and enhanced autophagy have been observed in HepG2 cells treated with acNPs.^372^ Zhang et al. designed two acidic nanoparticles based on PLGA and polylactic acid that effectively targeted macrophage lysosomes while rescuing lysosomal acidity.^373^ Therefore, restoring dysfunctional lysosomal acidification could be a viable strategy for eliminating cytosolic or lysosomal hydroxyapatite crystallites in chondrocytes.

Additionally, restoring LMP in chondrocytes could serve as a complementary therapeutic approach to mitigate lysosome-induced chondrocyte apoptosis, as such interventions alone may not address the underlying causes of LMP. While several studies have highlighted the protective roles of proteins such as Hsp70, LAMP-I, LAMP-II, Annexin A7, and ESCRT-III in maintaining lysosomal homeostasis,^331,333,374^ pharmacological agonists or recombinant proteins targeting these molecules for lysosome restoration remain paradoxically rare, except for a few studies investigating the use of Hsp70 or its agonist in lysosomal repair.^375^ Notably, exogenous administration of recombinant Hsp70 has been shown to regulate the activity of ASM on the lysosomes of primary fibroblasts, correcting the balance of lysosomal lipid composition and demonstrating the potential for alleviating LMP in chondrocytes.^376^ Further development of lysosome-targeting DDSs carrying Hsp70 or its agonists is warranted to increase lysosomal targeting, as nonselective upregulation of Hsp70 in the cytoplasm and membrane could disrupt normal physiological processes.^377^

#### Endoplasmic reticulum-targeting

**Fig. 8:**
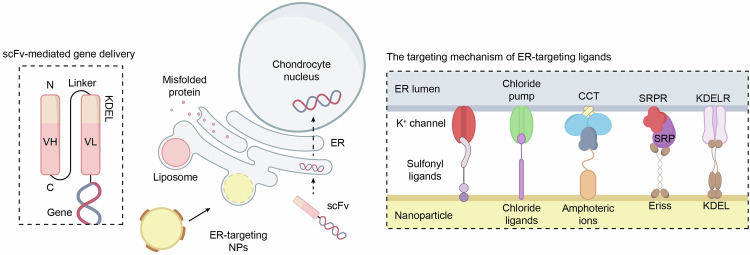
This diagram illustrates the sophisticated DDS designed to target the ER in chondrocytes, which is crucial for alleviating ER stress in OA. The left panel details the structure of ER-targeting scFv antibodies, which are conjugated with therapeutic genes to facilitate direct delivery to the ER, bypassing lysosomal degradation. The right panel outlines the targeting mechanism of ER-targeting ligands, which include sulfonyl ligands, chloride ligands, amphoteric ions, and nanoparticle ligands. These ligands interact with specific ER membrane proteins, such as the chloride pump, K^+^ channel, CCT, SRPR, and KDELR, to increase the ER-targeting ability of the DDS. The diagram highlights the potential of these ER-targeting strategies to restore chondrocyte ER homeostasis, thereby offering a promising approach for OA treatment. scFv single-chain fragment variable, CCT cytidylyltransferase, SPR signal recognition particle, SRPR signal recognition particle receptor, KDELR KDEL receptor

Therapeutic agents for alleviating ER stress have been widely discussed in the previous section. To overcome limitations in their therapeutic efficacy, such as issues related to biocompatibility, tissue distribution, or physicochemical properties, sophisticated DDSs should be developed to specifically target the chondrocyte ER, where therapeutic agents often struggle to reach. Current ER-targeting carriers can be broadly categorized into small molecules and targeted peptides (Fig. 8).^378^

Small molecules, including sulfonyl ligands, chloride ligands, and amphoteric ionic ligands, are commonly employed as ER-targetable fluorogenic probes and are often conjugated with fluorophores or ER-targeting carriers when modified with DDSs.^379^ Sulfonyl ligands, particularly glibenclamide, and its derivatives are widely recognized for their ER-targeting capabilities, which are mediated by their interaction with ATP-sensitive K^+^ channels located on the ER membrane via the cyclohexyl sulfonylurea moiety.^380^ Chloride ligands also offer an effective ER-targeting strategy by selectively binding to the chloride pump on the ER membrane.^379^ Additionally, a recently discovered zwitterionic aggregation-induced emission luminogen (AIEgen), which incorporates a sulfonyl group, demonstrates ER targetability, primarily attributed to electrostatic interactions between the amphoteric ion and the positively charged phosphocholine cytidylyltransferase on the ER membrane surface. This ER targetability is not solely derived from the sulfonyl group, as replacing the sulfonate moiety with p-bromobenzene shifts the targetability from the ER to the mitochondria. This observation underscores the prerequisite role of amphoteric properties in mediating ER targeting.^381,382^

ER-targeting peptides, including KDEL, KKXX, RXR (where X represents any amino acid), Eriss, and pardaxin,^379,383,384^ have been explored for their potential in targeting the ER. However, pardaxin is excluded from the subsequent discussion because of its proapoptotic effects. Both ER-targeting small molecules and peptide-conjugated nanomedicines remain the subject of ongoing investigation for their potential to restore chondrocyte ER homeostasis in OA treatment. For example, Yen et al. developed curcumin-encapsulated PLGA nanoparticles modified with KDEL on their surface. These nanoparticles inhibit the calcium-dependent interaction between UPR chaperones and misfolded proteins, thereby facilitating their escape from the ER lumen.^385^ In another study, Wang et al. designed sulfonyl ligand-modified PLGA nanoparticles loaded with an ER stress-inducing agent to promote apoptosis in cancer cells for melanoma therapy.^159^ Similar strategies could be adapted for OA treatment by replacing the encapsulated agent with ER stress inhibitors to restore chondrocyte ER homeostasis.

Driven by rapid advancements in organelle-targeting strategies and biomedical technologies, novel approaches such as liposome fusion and monoclonal antibodies have emerged as promising solutions for ER targeting. Liposomes, as self-assembled phospholipid carriers, can be structurally modified to mimic the ER membrane, thereby increasing their ER-targeting potential. Alternatively, ER-targeting ligands can be incorporated onto the surface of liposomes to improve their targeting specificity.^386,387^ Pollock et al. developed an ER-targeting liposome with a phospholipid composition resembling that of a rough ER membrane that was purified from rat liver. The study demonstrated that the liposome utilizes scavenger and low-density lipoprotein receptor-mediated endocytosis, followed by caveolin- and microtubule-dependent trafficking to the ER. Once at the ER, the liposome fuses with the ER membrane, enabling continuous cargo release.^388^ Yuan et al. designed pardaxin-modified liposomes to induce apoptosis in cancer cells. Compared with nonmodified liposomes, these liposomes exhibited enhanced ER colocalization in cancer cells at 8 h,^389^ suggesting that similar liposome-based strategies—the replacement of pardaxin with nontoxic ER-targeting ligands—could be effective in restoring chondrocyte ER homeostasis for OA treatment.

In addition to restoring ER function, gene therapy holds promise for OA treatment by delivering genetic materials that promote articular protection to chondrocytes.^390^ However, conventional gene delivery methods typically follow the endosome‒lysosome route, leading to the degradation of gene payloads and diminished therapeutic efficacy.^391^ ER-targetable monoclonal antibodies offer a potential solution to this issue by circumventing lysosomal degradation while simultaneously delivering therapeutic genes directly to the ER, near the nucleus, where they are protected from degradation and can be efficiently delivered to the nucleus.^392,393^ These ER-targeting monoclonal antibodies are often designed via a single-chain fragment variable approach, in which the N-terminal single-chain fragment variable domain, which contains a target site-specific signal sequence, is conjugated with therapeutic genes, while the C-terminal ER-targeting signal KDEL is incorporated.^379^ However, the biosafety and efficacy of such ER-targeting monoclonal antibodies in restoring chondrocyte ER homeostasis require further validation.

### Other osteoarthritis therapies

Emerging therapeutic innovations are reshaping OA management through novel biological strategies. This section explores cell-based immunotherapies such as mesenchymal stem cells (MSCs), which combine immunomodulatory effects with anti-inflammatory effects, and engineered extracellular vesicles (EVs) designed for targeted therapeutic delivery and biomarker discovery. These approaches highlight the diversification of OA interventions addressing both immune dysregulation and tissue repair.

#### Cell-based immunotherapy

Given that cytokines and chemokines in the OA microenvironment recruit activated neutrophils and monocytes into the knee joint, where the recruited immune cells secrete proinflammatory mediators, which in turn recruit additional immune cells (e.g., macrophages and effector T cells), creating a vicious cycle,^394^ immunotherapy has emerged as a promising therapeutic strategy for OA treatment. Current immunotherapeutic approaches for OA are categorized into two primary modalities: conventional and biologic therapies and cell-based therapies.^395^ Conventional and biologic therapies, which include anticytokine strategies, have been extensively delineated in the preceding sections. In contrast, cell-based therapies constitute a distinct therapeutic approach, with MSCs being the most prevalent modality for OA treatment.^396^ MSCs promote chondrogenesis through their capacity for chondrocyte differentiation, coupled with anti-inflammatory and immunomodulatory effects that synergistically suppress pathological inflammation and rebalance dysregulated immune responses,^397^ including promoting the transformation of proinflammatory M1 macrophages into anti-inflammatory M2 macrophages, inhibiting T-cell proliferation and activity, promoting the development of anti-inflammatory regulatory T (T_reg_) cells, and reducing B-cell proliferation and differentiation, thereby decreasing antibody production.^395^ Clinical trials have shown that intra-articular injection of MSCs into the knee joints of OA patients results in significant results in cartilage repair and enhanced chondrogenesis with recoverable mild to moderate side effects.^397^

Macrophages are highly plastic immune cells that serve as powerful producers of cytokines and chemokines in inflamed joints. Targeting macrophages as a therapeutic approach holds promise for the treatment of OA.^398^ A preclinical study demonstrated that intra-articular injection of genetically engineered macrophages locked in the M2 phenotype has anti-inflammatory and pro-regenerative effects in OA rats.^399^ Moreover, the ability of Treg cells, which have the capacity to mediate immune responses and suppress inflammation, is considered a promising therapeutic strategy for OA, as autoantibodies against type II collagen are detected in nearly 50% of OA patients.^400^ Sohn et al. designed nanoparticles loaded with antigens (e.g., collagen II) and rapamycin, which facilitate tolerogenic dendritic cell responses. Upon uptake of nanoparticles, dendritic cells differentiate into tolerogenic DCs and induce antigen-specific immunomodulatory responses (such as the induction of antigen-specific regulatory (T_reg)_ cells), thereby modulating the immune response against type II collagen. Intra-articular injection of the nanoparticles inhibited cartilage degradation and relieved pain in OA model mice.^401^

#### Extracellular vesicles in osteoarthritis treatment

EVs have emerged as promising therapeutic candidates for OA, offering novel potential to address the persistent challenge of disease-modifying therapies in joint degeneration. EVs are generated primarily through endosomal pathways, wherein early endosomes undergo inward membrane budding to form numerous intraluminal vesicles. These late endosomes, known as multivesicular bodies, subsequently fuse with the plasma membrane, releasing their internal intraluminal vesicles into the extracellular space as EVs.^402,403^ Characterized by their lipid bilayer membrane structure, EVs differ fundamentally from conventional cell-based therapies in that they retain donor cell-derived surface markers, circumvent ethical concerns, and exhibit reduced risks of immune rejection and tumorigenesis.^404^ Their therapeutic utility is further enhanced by their inherent targeting capabilities and engineerable cargo-loading properties, positioning them as versatile platforms for OA intervention.

Conventional imaging techniques primarily detect OA at advanced stages, necessitating biomarkers for early diagnosis.^405^ EVs mediate intercellular crosstalk through the selective transfer of bioactive lipids, proteins, and RNA species, including mRNAs, microRNAs, and long noncoding RNAs, demonstrating clinically exploitable diagnostic potential via OA-specific molecular cargo.^405,406^ Recent studies have demonstrated s-associated biomarker alterations in OA pathophysiology. For example, plasma EV miR-193b-3p levels are considerably lower than those in healthy controls, whereas synovial fluid EV miR-182-5p levels progressively decrease with disease severity. Notably, the synovial fluid EV-derived lncRNA PCGEM1 was markedly elevated in late-stage OA patients compared with early-stage OA patients, and the serum EV circ_008365 was downregulated in OA patients.^403,407,408^ These stage-specific EV biomarker modifications may hold diagnostic potential for OA.

Preclinical studies have demonstrated that native EVs derived from MSCs, including bone marrow-derived MSCs (BMSCs), adipose-derived MSCs (AMSCs), synovial MSCs (SMSCs), and embryonic MSCs (EMSCs), attenuate cartilage degradation and suppress inflammatory responses, highlighting their therapeutic potential for OA treatment.^407^ For example, BMSCs were the first MSCs applied in OA therapeutics because of their clinical feasibility in isolation, proliferation, and cryopreservation.^409^ BMSC-derived EVs were found to suppress the expression of catabolic markers (ADAMTS5 and MMP-13) and proinflammatory cytokines (IL-1β, IL-6, and TNF-α) while increasing the expression of anabolic markers (COL II and aggrecan) in vitro and to mitigate cartilage degeneration and joint pathology in OA rat models.^403,407^ However, BMSC-based therapies have intrinsic limitations, including age-dependent decreases in stem cell quantity/differentiation capacity, invasive extraction procedures causing donor morbidity, and restricted bone marrow yields.^410^ In contrast to BMSCs, AMSCs exhibit superior tissue accessibility due to the ubiquitous distribution of adipose depots. AMSC-derived EVs (AMSC-EVs) have robust anti-OA effects through the downregulation of proinflammatory cytokines (IL-1β, TNF-α, and IL-6), the upregulation of anti-inflammatory IL-10, and the restoration of collagen II synthesis in OA chondrocytes.^403,407^ Intra-articular delivery of AMSC-EVs substantially attenuated OA progression in OA rats, reducing cartilage degeneration while preserving subchondral bone architecture.^407^ Similarly, EVs derived from SMSCs and EMSCs also exhibited significant anti-OA effects in both in vitro and in vivo models.^407,411^

EVs inherently encapsulate proteins, lipids, and nucleic acids, demonstrating exceptional biocompatibility and drug-loading capacity that positions them as potent therapeutic carriers.^412^ These vesicles deliver payloads through two complementary mechanisms: cellular internalization via clathrin-dependent or clathrin-independent endocytosis, phagocytosis, or micropinocytosis, and extracellular engagement through ligand‒receptor interactions that activate downstream signaling cascades to achieve therapeutic effects.^413^ However, naturally derived EVs exhibit limited targetability and inherent heterogeneity, even when they originate from an identical recipient cell type.^414^ To overcome these challenges, surface modification of EVs for targeted delivery, including genetic modification of donor cells to produce EVs with specific surface proteins or direct conjugation of targeting moieties to the EV surface, is employed to increase tissue-specific delivery precision, therapeutic bioavailability, and biosafety while minimizing immunogenicity and off-target toxicity.^403,406^ In OA treatment, EVs are commonly modified with chondrocyte affinity peptide (CAP) to enable precise chondrocyte targeting.^415^ Therapeutic cargo loading primarily utilizes two strategies: genetic modification of parent cells to overexpress native or engineered therapeutic molecules and exogenous methods such as passive incubation with drugs, electrostatic complexation using transfection reagents for nucleic acids, or physical techniques such as electroporation and sonication to transiently permeabilize EV membranes.^415^ Furthermore, hybrid EV-liposome systems have been developed to synergistically mitigate liposomal toxicity while overcoming the limited drug-loading capacity of natural EVs, offering a dual-functional platform for enhanced therapeutic delivery.^415^ Liang et al. developed chondrocyte-targeting EVs by transfecting dendritic cells with plasmids encoding a fusion protein combining CAP and the transmembrane protein lysosome-associated membrane glycoprotein 2b (Lamp2b), enabling EV surface display of CAP-Lamp2b. Subsequent electroporation transiently permeabilized EV membranes to encapsulate miR-140, a miRNA involved in cartilage development and homeostasis. Intra-articular administration of these bioengineered EVs in a rat OA model substantially alleviated OA progression.^416^ Xu et al. engineered EVs through surface modification with a fusion protein combining the MSC-binding peptide E7 and Lamp2b to achieve SMSC targeting, accompanied by electroporation-mediated encapsulation of kartogenin to induce SMSC differentiation into chondrocytes. This dual-functional approach demonstrated enhanced chondrogenesis both in vitro and in vivo.^417^

Additionally, the anionic lipid bilayer of native EVs impedes penetration through the negatively charged cartilage matrix, substantially limiting therapeutic efficacy in the deep zones of cartilage during OA treatment.^415^ Bajpayee et al. engineered charge-reversible EVs by anchoring arginine-rich cationic motifs (CPCs + 14Rs) to anionic EV surfaces via pH-responsive switches, shifting the zeta potential from −25.4 ± 1.1 mV to −2.5 ± 1.0 mV. In destabilized medial meniscus-induced OA mice, intra-articular administration of these modified EVs enhanced cartilage penetration into deep zones, effectively alleviating OA pathological progression.^418^

## Conclusion and perspective

Current therapeutic strategies for symptomatic relief and pathological management of OA are insufficient. Traditional pharmacological treatments and surgical interventions are frequently associated with adverse side effects and often fail to provide meaningful pain relief or mitigate the catabolic processes driven by inflammation and dysfunction of cellular organelles. Consequently, a deeper understanding of the mechanisms underlying OA pain, coupled with the inflammation and destabilization of cellular organelles that occur during disease progression, offers a promising avenue for identifying novel therapeutic targets.

A key objective in OA pain management is to interrupt pain signal transmission before it reaches the brain and is perceived as pain. Pharmacological blockade of ion channels in peripheral nociceptors, which detect noxious stimuli, and inhibition of NGF, which sensitizes these ion channels, are promising approaches for attenuating OA pain. Additionally, targeting neurotransmitters involved in the synaptic transmission of nociceptive signals may further contribute to pain relief. Several agents targeting these pathways have demonstrated promising results in preclinical animal models. However, their clinical efficacy and feasibility for OA pain management require validation through rigorous clinical trials.

Furthermore, the inflammatory cytokines accompanying dysfunctional chondrocyte organelles exacerbate catabolic processes in the knee joint. Therapeutic agents targeting key components of the inflammatory signaling pathways involved in OA progression may surpass conventional anti-inflammatory drugs, offering greater specificity for OA treatment. In addition, dysfunctional chondrocyte organelles contribute to cartilage destruction through chondrocyte apoptosis, and pharmacological agents aimed at restoring organelle homeostasis in chondrocytes present an alternative approach for OA therapy. However, therapeutic intervention for dysfunctional organelles in OA treatment is still in its infancy, and their therapeutic efficacy requires further investigation.

Moreover, advancement of sophisticated DDSs for organelle-targeting therapeutics is essential to address the challenges associated with biological membrane permeability, a barrier that most of these agents struggle to overcome. Concurrently, these systems must enhance therapeutic efficacy, optimize in vivo distribution, and minimize toxicity. Given the dearth of studies on organelle-targeting DDSs in OA, methodologies derived from treatments for other pathological conditions may provide valuable frameworks for future research aimed at restoring the functionality of dysfunctional chondrocyte organelles.

The primary limitation of current studies on OA is that most of the therapeutic strategies described are based on targets identified through the exploration of OA-related mechanisms, offering a strong theoretical foundation but lacking experimental validation in OA models. This is particularly evident in treatments aimed at addressing imbalances in cellular organelles within osteoarthritic joints. For example, drugs designed to restore mitochondrial homeostasis have been evaluated predominantly in preclinical OA studies. However, the side effects of the majority of these drugs remain unknown, and their efficacy has been confirmed largely only in OA cellular models, with insufficient data on their in vivo therapeutic effects and optimal drug delivery formulations. Furthermore, the efficacy of pharmacological agents that target lysosomes and the ER has, in most cases, not been verified in OA models.

Given the challenges of delivering drugs to organelles within chondrocytes, the previous section detailed the design of organelle-targeted DDSs to enhance in vivo efficacy. However, these designs are primarily based on mechanistic considerations of organelle targeting and lack experimental validation specific to chondrocytes. To guide future OA treatment, a roadmap is proposed for the clinical translation of DDSs aimed at repairing OA-affected organelles. This involves refining in vivo drug formulations and conducting pharmacological and toxicological studies to elucidate drug behavior, including the half-life, efficacy, and side effects, in OA therapy.

To confirm that therapeutic agents target the intended sites, relevant biomarkers, such as organelle-related markers (e.g., ΔΨm, ATP, mtDNA, ROS, UPR chaperones, cathepsins, RUNX2, and type X collagen), should be monitored postadministration. Efficacy and toxicity data from animal models should be submitted to regulatory authorities (e.g., the FDA). Subsequently, phase 0 trials using microdoses with probes in healthy volunteers can leverage imaging to verify drug localization, followed by phase 1 trials to establish safe dosages for OA patients, monitor organelle biomarkers in synovial fluid, and evaluate overall therapeutic effects on OA.
